# ETV4-Mediated PD-L1 Upregulation Promotes Immune Evasion and Predicts Poor Immunotherapy Response in Melanoma

**DOI:** 10.32604/or.2025.070180

**Published:** 2025-12-30

**Authors:** Tao Zhu, Taofeng Wei, Mingdong Yang, Junjun Xu, Huifang Jiang, Wei He, Juyan Zheng, Haibin Dai

**Affiliations:** 1 Department of Pharmacy, The Second Affiliated Hospital, Zhejiang University School of Medicine, Hangzhou, 310009, China; 2 Center for Clinical Pharmacy, Cancer Center, Department of Pharmacy, Zhejiang Provincial People’s Hospital (Affiliated People’s Hospital), Hangzhou Medical College, Hangzhou, 310014, China

**Keywords:** Melanoma, immune evasion, ETS transcription factors, E-twenty-six variant 4, immunotherapy

## Abstract

**Background:**

Aberrant expression of transcription factors (TFs) is a key mechanism mediating tumor immune escape and therapeutic resistance. The involvement of E26 transformation-specific (ETS) family of TFs in immune regulation is not fully understood. The study aimed to elucidate the function of E-twenty-six variant 4 (ETV4) in tumor immune evasion and its potential as a predictive biomarker for immunotherapy in melanoma.

**Methods:**

The expression patterns of ETS family TFs were analyzed in melanoma and hepatocellular carcinoma (HCC). Single-cell RNA sequencing (scRNA-seq) was used to dissect the cellular expression and function of ETV4 in the tumor microenvironment. Functional studies and murine models were employed to investigate the role of ETV4 in T cell-mediated tumor killing and tumor growth. The correlation between ETV4 expression level and patient responsiveness to programmed cell death protein 1 (PD-1) blockade therapy was evaluated.

**Results:**

TFs in the ETS family were found to effectively stratify melanoma and HCC patients into prognostic subgroups. In melanoma, the polyoma enhancer activator 3 (PEA3) subfamily, particularly ETV4 and ETV5, showed a negative correlation with immune infiltration. scRNA-seq analysis showed that ETV4 is preferentially expressed in melanoma cells and involves in mediating tumor-immunocyte interactions. Functional studies demonstrated that ETV4 impairs T cell-mediated tumor killing by transcriptionally upregulating programmed death-ligand 1 (PD-L1). In immunocompetent murine models, ETV4 downregulation significantly suppressed tumor growth. Furthermore, high ETV4 expression correlated with poor responses to anti-PD-1 therapy.

**Conclusion:**

Our findings identify ETV4 as a key transcriptional regulator of immune evasion in melanoma by controlling PD-L1 expression. ETV4 may act as a predictive biomarker for immunotherapy outcomes.

## 1 Introduction

Tumor immune evasion represents an essential strategy acquired by tumor cells to enable them to proliferate and spread while avoiding immune surveillance and attack. This evasion is facilitated through multiple mechanisms, including the elevated expression of immune checkpoints such as programmed death-ligand 1 (PD-L1), defective antigen presentation process, and the infiltration of immune-suppressing cells, such as myeloid-derived suppressor cells (MDSCs) and regulatory T cells (Tregs), into the tumor microenvironment (TME) [1–3]. The emergence of immunotherapy marks a significant breakthrough for the treatment of cancer, providing remarkable clinical benefits for certain patients [4]. However, despite the proven effectiveness of immune checkpoint inhibitors (ICIs), many patients still experience primary or acquired resistance, which is closely associated with tumor immune evasion [5].

Aberrant expression of TFs is a well-established mechanism by which tumors achieve immune evasion and develop resistance to immunotherapy. Accumulating transcription factors involved in these processes have been identified, such as STAT3 [6,7], HIF-1α [8], ATF3 [9], ZEB1 [10] and TCF4 [11]. For example, ATF3 has been shown to directly enhance PD-L1 transcription in tumor cells, which correlates with programmed cell death protein 1 (PD-1) blockade therapy efficacy [9]. Additionally, ATF3 downregulates CH25H, a critical regulator of T cell trogocytosis and survival, in intra-tumoral cytotoxic T cells, resulting in their decreased viability and compromised anti-tumor immune responses [12]. Furthermore, changes in transcriptional profiling are crucial for the acquisition and maintenance of cancer hallmarks. The family of E26 transformation-specific (ETS) transcription factors, composed of 28 members that share a conserved DNA-binding domain, exhibits dysregulated activity that contributes to various cancer hallmarks, such as unchecked proliferation, metastasis, genomic instability, and deregulated cellular metabolism [13]. In addition to their role in cancer progression, studies have revealed that ETS family members are crucial for regulating immune cell development and function [14]. For instance, ETS1 has been found to suppress cytotoxic T cell differentiation [15]. Moreover, emerging evidence suggests that ETS family proteins in tumor cells also regulate tumor immunity [16,17], further highlighting their influence on both tumor growth and the immune response. So far, the role of most ETS transcription factors in tumor immunity, as well as their potential as indicators of response to immunotherapy, remains unclear.

Here, we analyzed expression profiles of ETS transcription factors in melanoma and HCC. Melanoma and hepatocellular carcinoma represent two distinct tumor immune microenvironments. Melanoma is typically considered an immune “hot” tumor, characterized by a T cell-rich microenvironment and responsiveness ICI therapy [18], whereas HCC exhibits features of an immune “cold” tumor, with limited effector T cell infiltration and a more immunosuppressive microenvironment [19]. By selecting these two tumor types, we aimed to investigate whether members of the ETS family exhibit conserved or context-dependent roles in regulating tumor immunity. Specifically, we sought to determine whether the expression patterns of the 28 ETS family members are associated with overall survival and tumor immune landscapes, and to determine the potential involvement of the PEA3 subfamily members in shaping immune infiltration. Furthermore, we explored the preferential expression of ETV4 in melanoma cells within the TME and its possible involvement in mediating crosstalk between tumor cells and infiltrating immunocytes. Finally, we set out to elucidate the roles of ETV4 in regulating immune evasion and immunotherapy response.

## 2 Materials and Methods

### 2.1 Data Acquisition

The Cancer Genome Atlas (TCGA) bulk RNA sequencing data of the skin cutaneous melanoma (SKCM) and liver hepatocellular carcinoma (LIHC) were retrieved from University of California, Santa Cruz (UCSC) Xena (https://xena.ucsc.edu/). Corresponding clinical data for the patients in these cohorts was obtained from TCGA. Cases lacking complete survival data or those whose overall survival was <30 days were excluded. Two scRNA-seq datasets of melanoma (GSE72056 and GSE115978) were retrieved from the Tumor Immune Single-cell Hub (TISCH) database (http://tisch.comp-genomics.org/).

### 2.2 Consensus Clustering Analysis

To identify patient clusters according to the expression patterns of the 28 ETS TFs, consensus clustering analysis was performed with the ‘ConsensusClusterPlus’ R package (version 1.66.0). The optimal cluster count was identified by evaluating the cumulative distribution function curve and consensus matrices.

### 2.3 Functional Enrichment Analysis

Gene set variation analysis (GSVA) was employed to evaluate the activity of the 50 hallmark pathways as defined inthe Molecular Signatures Database (MSigDB, https://www.gsea-msigdb.org/gsea/msigdb, accessed on 01 September 2025) in each patient, using the ‘GSVA’ R package (1.50.0). Pathway enrichment analyses for Kyoto Encyclopedia of Genes and Genomes (KEGG) and Gene Ontology (GO) were conducted utilizing the gene set enrichment analysis (GSEA) algorithm within the ‘clusterProfiler’ R package (4.10.1). To investigate the immune infiltration landscape within the TME, the ESTIMATE algorithm was applied. Infiltration of diverse immune cell types was analyzed with the ‘GSVA’ package, using gene sets representing 28 immune cell subtypes obtained from an integrated repository portal for Tumor-Immune System Interactions (TISIDB) (http://cis.hku.hk/TISIDB/, accessed on 01 September 2025).

### 2.4 Hypergeometric Optimization of Motif Enrichment (HOMER) Analysis

We conducted *de novo* and known motif enrichment analyses using the HOMER software (version 5.1, San Diego, CA, USA). Promoter regions of differentially expressed genes (DEGs) were compared against a collection of background regions to identify statistically overrepresented DNA sequence motifs. The hypergeometric test was utilized for enrichment scoring.

### 2.5 Single-Cell RNA Sequencing (ScRNA-Seq) Analysis

ScRNA-seq data was analyzed with the ‘Seurat’ R package (5.0.1). Gene expression matrices were converted to Seurat objects. Cells expressing fewer than 200 genes and those with mitochondrial gene expression exceeding 5% were filtered out due to the low-quality. We normalized and scaled the expression matrix using the default parameters, and then performed dimensionality reduction via principal component analysis. After that, unsupervised clustering was conducted, with discrete cell clusters identified via the FindClusters function and visualized through t-distributed stochastic neighbor embedding (t-SNE). Canonical marker genes were utilized to annotate cell clusters. Melanoma cells were identified using MIA and TYR cell markers [20]. For sub-clustering of melanoma cells, the raw melanoma cell expression data were extracted and reanalyzed using the same procedures. The ‘CellChat’ R package (version 1.6.1) was employed for analysis of cell-cell communication.

### 2.6 Cell Culture and Transfection

Human melanoma cell lines A375 and SK-MEL-28 were maintained in DMEM (C11995500BT, Gibco, Waltham, MA, USA) supplemented with 10% fetal bovine serum (FBS) (FSP500, ExCell Bio, Suzhou, China). Murine MC38 cells were maintained in RPMI 1640 medium (C11875500BT, Gibco, Waltham, MA, USA) containing 10% FBS. These cell lines were purchased from Meisen (Hangzhou, China) and verified via short tandem repeat (STR) profiling. Prior to use, each cell line was tested and found to be free of mycoplasma contamination. All these cells were maintained in a humidified atmosphere at 37°C with 5% CO_2_. Transfections with small interfering RNA (siRNA) or plasmids were performed using the jetPRIME reagent (101000046, Polyplus, Illkirch, France). The siRNA targeting ETV4 was acquired from GenePharma (Shanghai, China). The human ETV4-expressing plasmid (EX-T8074-M02) was obtained from GeneCopoeia (Guangzhou, China). The sequences of the siRNAs were as follows: ETV4 siRNA-1, 5^′^-GGGCAGAGCAACGGAAUUU-3^′^; ETV4 siRNA-2, 5^′^-GAAUGGAGUUCAAGCUCAU-3^′^; and control siRNA, 5^′^-UUCUCCGAACGUGUCACGU-3^′^.

### 2.7 Quantitative Reverse Transcription Polymerase Chain Reaction (qRT-PCR)

Total RNA was extracted from A375 and SK-MEL-28 cells using RNAiso Plus (9108, TaKaRa, Kusatsu, Japan), and reverse transcription was conducted by using the PrimeScript RT Reagent Kit (RR047A, TaKaRa, Kusatsu, Japan). The resulting complementary DNA (cDNA) was thenanalyzed by qRT-PCR using the TB Green Premix Ex Taq II reagent (RR420A, TaKaRa, Kusatsu, Japan) on the LightCycler 480 System (Roche, Mannheim, Germany) for 40 cycles. Relative gene expression was quantified by using the 2^−ΔΔCT^ method. The sequences of primer used in this study were all sourced from PrimerBank (https://pga.mgh.harvard.edu/primerbank/index.html, accessed on 01 September 2025) and synthesized by Tsingke Biotechnology (Beijing, China). The sequences are provided in Supplementary Table S1.

### 2.8 Dual Luciferase Reporter Assay

A375 and SK-MEL-28 cells were seeded in 12-well plates. Once reaching 60%–70% confluence, they were transfected with either ETV4-targeting siRNA or ETV4-expressing plasmids. After 24 h, the pGL3-CD274 plasmid or the control pGL3-basic vector was transfected, along with the pRL-TK plasmid for normalization. Following an additional 24 h of culture, the activity of the promoter was assessed by measuring luciferase activity by using the Dual-Luciferase Reporter Assay System (E1910, Promega, Madison, WI, USA). The pGL3-CD274 plasmid, which contains the promoter region of CD274 (−2000 to +100 bp), was obtained from YouBio (Changsha, China).

### 2.9 Flow Cytometry Analysis

For the measurement of cell surface expression of PD-L1, we used anti-CD274-PE (329706, 1:21, BioLegend, San Diego, CA, USA) for staining and cell samples were assessed through a CytoFLEX flow cytometer (Beckman Coulter, Indianapolis, IN, USA). In brief, A375 and SK-MEL-28 cells were trypsinized with 0.25% trypsin (25200072, Thermo Fisher Scientific, Waltham, MA, USA) to obtain a single-cell suspension and incubated with Fc receptor blocking solution (422301, BioLegend, San Diego, CA, USA) for 5 min. Subsequently, 100 µL of the cell suspension was stained with 5 µL of anti-CD274-PE or an isotype control for 20 min. After washing, the stained cells were subjected to flow cytometry analysis. Data were further analyzed using FlowJo 10.0 software (BD Biosciences, Ashland, OR, USA).

### 2.10 Western Blot Assay

Western blotting assays were performed as previously described [21]. Briefly, A375 and SK-MEL-28 cells were lysed in RIPA buffer (Beyotime, Shanghai, China) containing 1% protease inhibitors (Beyotime, Shanghai, China), and the lysates were centrifuged for 10 min at 12,000× *g*, the supernatant was collected. Protein concentrations were determined using a bicinchoninic acid assay kit (P0012S, Beyotime, Shanghai, China). After that, equal amounts of protein samples were separated by SDS-polyacrylamide gel electrophoresis (SDS-PAGE) and then transferred onto polyvinylidene difluoride (PVDF) membranes (Millipore, Darmstadt, Germany). Membrane blocking was performed with 5% skim milk for 1 h at room temperature. Subsequently, the membranes were incubated with primary antibodies at 4°C overnight. After washing, membranes were incubated with corresponding secondary antibodies for 1 h at room temperature. Protein bands were visualized using an Omni-ECL kit (SQ201, Epizyme, Shanghai, China) and imaged with a chemiluminescence system (Tanon, Shanghai, China). The primary antibodies used were as follows: anti-ETV4 (sc-113, 1:1000, Santa Cruz, Dallas, TX, USA), anti-PD-L1 (ab213524, 1:1000, Abcam, Cambridge, Cambridgeshire, UK), anti-MHC-I (ab134189, 1:2000, Abcam, Cambridge, Cambridgeshire, UK), and anti-β-actin (sc-47778, 1:1000, Santa Cruz, Dallas, TX, USA).

### 2.11 Immunofluorescence Analysis

Cells with ETV4 knockdown via siRNA were seeded onto 35 mm confocal dishes at a density of 3 × 10^5^ cells in 1 mL medium per dish. The following day, the cells were fixed with 4% paraformaldehyde, blocked with immunofluorescence blocking solution, and incubated with anti-ETV4 (sc-113, 1:100, Santa Cruz, Dallas, TX, USA) and anti-PD-L1 (ab213524, 1:500, Abcam, Cambridge, Cambridgeshire, UK) at 4°C overnight. After washing, the cells were treated with corresponding fluorescently labeled secondary antibodies including Alexa Fluor 488 goat anti-rabbit IgG H&L (ab150077, 1:300, Abcam, Cambridge, Cambridgeshire, UK) and Alexa Fluor 647 goat anti-mouse IgG H&L (ab150115, 1:300, Abcam), and nuclei were stained with DAPI (Beyotime, Shanghai, China). Immunofluorescence images were captured using a laser confocal microscope (LSM 900, ZEISS, Oberkochen, Baden-Württemberg, Germany) and analyzed with ZEN imaging software (version 3.4, ZEISS, Oberkochen, Baden-Württemberg, Germany).

### 2.12 T Cell-Mediated Tumor Cell Killing Assay

Peripheral blood mononuclear cells (PBMCs) were isolated from the peripheral blood of normal subjects (n = 3) recruited at the Second Affiliated Hospital of Zhejiang University School of Medicine using SepMate tubes (STEMCELL Technologies, Vancouver, Canada). CD8^+^ T cells were enriched via magnetic cell sorting (MACS) using the EasySep Human CD8 Positive Selection Kit (17853, STEMCELL Technologies, Vancouver, Canada). The enriched T cells were maintained in RPMI 1640 containing 10% FBS, recombinant human IL-2 (200-02, 1000 U/mL, PeproTech, Cranbury, NJ, USA), anti-CD3 antibody (300438, 1:1000, BioLegend, San Diego, CA, USA), and anti-CD28 antibody (302934, 1:2000, BioLegend, San Diego, CA, USA). After 5–7 days of culture, CD8^+^ T cells were cocultured with tumor cells at an initial ratio of 3:1 for 48 h. We used 0.1% crystal violet to stain the viable tumor cells. This study was approved by the Human Research Ethics Committee of the Second Affiliated Hospital of Zhejiang University School of Medicine (IRB-2022-0693). All subjects provided written informed consent.

### 2.13 Animal Studies

Lentiviral vectors carrying the corresponding shRNA were obtained from Genechem (Shanghai, China) and transfected into cells, followed by selection with puromycin to generate stable MC38 cell line. Four-to-six weeks old female BALB/c nude mice and C57BL/6 mice were purchased from SLAC (Shanghai, China) and housed in specific pathogen-free conditions under a 12-h light/dark cycle with free access to food and water. Mice were randomly assigned to two groups (n = 6 each group). A suspension of 5 × 10^5^ MC38 cells, either expressing Etv4 shRNA or a control, was prepared in 100 μL of medium and injected subcutaneously into the flanks of the mice. Every 2–3 days the tumor volumes in each group were measured using a digital caliper and calculated as (length × width^2^)/2, with operators blinded to group allocation. All animal experiments were carried out according to the guidelines approved by the Animal Experimentation Ethics Committee (AIRB-2022-1053). The shRNA sequences targeting Etv4 were synthesized by ZKbiotech (Guangzhou, China) and listed in Supplementary Table S2.

### 2.14 Statistical Analysis

Statistical analyses were performed using R (4.3.1) or GraphPad Prism software (9.5.1, San Diego, CA, USA). Bioinformatic data analysis and visualization were performed using R (4.3.1). Quantitative data are presented as mean ± standard deviation (SD). Unpaired Student’s *t*-tests were used to assess differences between two groups. Survival differences were evaluated using the Kaplan-Meier (KM) method and log-rank tests. *p* < 0.05 was considered statistically significant.

## 3 Results

### 3.1 Expression Pattern of ETS Transcription Factors Dichotomizes Melanoma Patients and Predicts Distinct Overall Survival Outcomes

**Figure 1:**
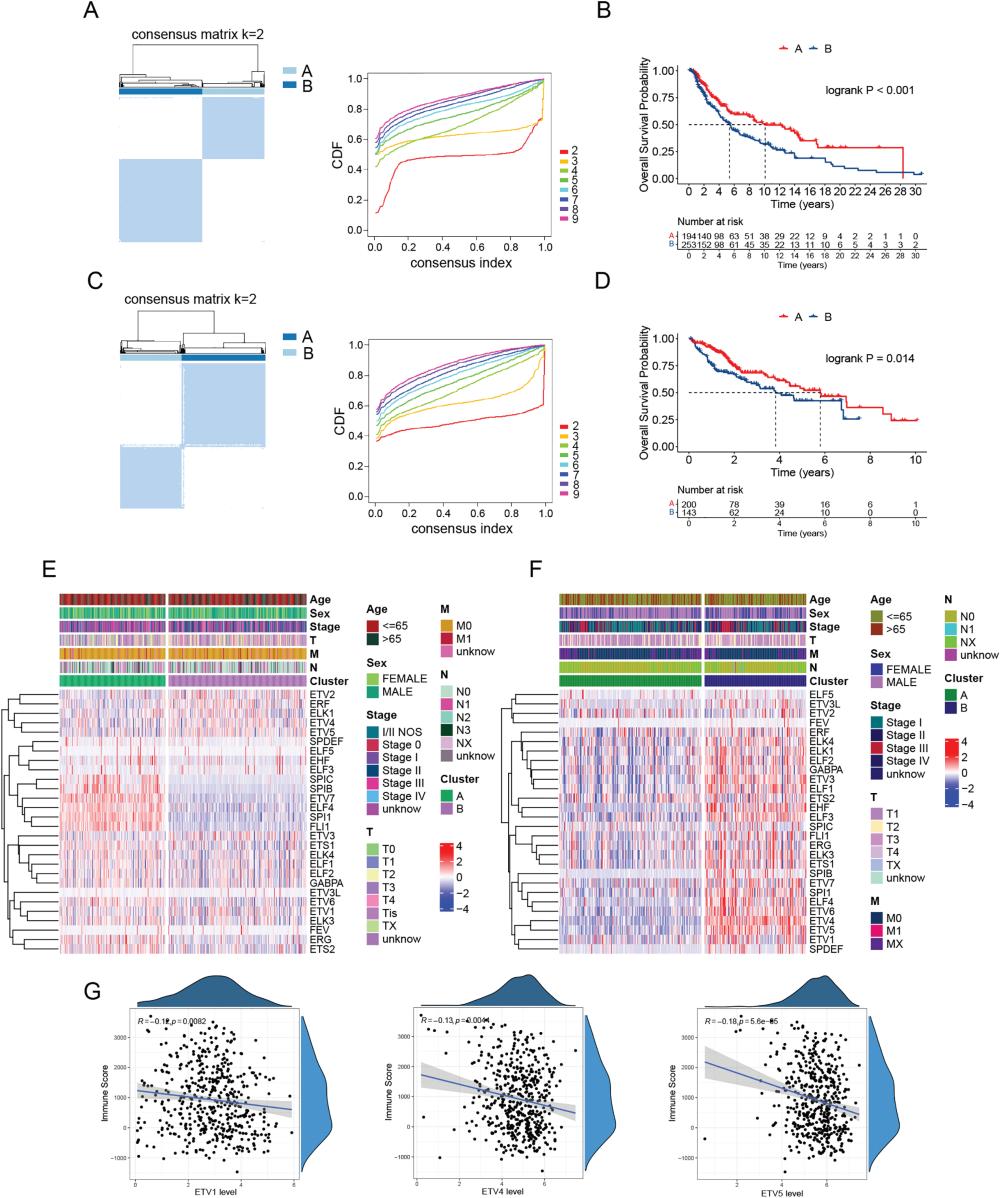
ETS transcription factor expression patterns dichotomize patients and predict survival in melanoma and HCC. (**A**) Consensus matrix heatmap (left) and the cumulative distribution function (CDF, right) demonstrating the optimal categorization of melanoma in the TCGA cohort. (**B**) Kaplan-Meier curves for overall survival in the two melanoma clusters. (**C**) Consensus matrix heatmap (left) and CDF (right) demonstrating the optimal categorization of HCC in the TCGA cohort. (**D**) Kaplan-Meier curves for overall survival in the two HCC clusters. (**E**,**F**) Heatmaps showing expression of the 28 ETS transcription factors and clinical feature distribution in melanoma clusters (**E**) and HCC clusters (**F**). (**G**) Correlation analysis of the expression levels of the PEA3 subfamily members (ETV1, ETV4, and ETV5) with immune scores in the TCGA melanoma dataset

The majority of the 28 TFs in the ETS family (Supplementary Table S3) have been implicated in tumor progression. Previous studies have identified multiple immune subtypes, and transcription factor expression can serve to define cancer subtypes [22,23]. Based on this, we hypothesized that the ETS transcription factor signature may have the potential to classify cancer subtypes. Consensus clustering analysis of the TCGA melanoma dataset, a tumor type commonly referred to as “hot” [18], based on the expression of 28 ETS transcription factors, revealed two distinct clusters: Cluster A and Cluster B (Fig. 1A). Survival analysis showed that Cluster A patients had significantly longer overall survival compared to Cluster B patients (Fig. 1B). We investigated whether a similar pattern is present in HCC, an immunologically “cold” tumor type [24]. Likewise, two clusters were identified in the TCGA HCC dataset based on the expression of the 28 TFs (Fig. 1C), with Cluster B associated with poorer overall survival (Fig. 1D). We compared expression of these TFs between the patient clusters. In melanoma, several ETS TFs, including FLI1, SPI1, ELF4, ETV7, SPIB, and SPIC, are expressed at higher levels in Cluster A patients, whereas ETV5, ETV4, ELK1, ERF, and ETV2 showed increased expression in Cluster B patients (Fig. 1E). In contrast, majority of the ETS TFs were expressed at higher levels in Cluster B in HCC (Fig. 1F). Melanoma arises in the skin, typically exhibiting a “hot” tumor phenotype enriched in cytotoxic T cells [18], whereas HCC originates in the liver, a tolerogenic organ with high frequencies of immunosuppressive cells [19]. The opposite expression patterns of most ETS family members observed in HCC and melanoma clusters likely reflect fundamental differences in their tumor immune microenvironments, transcriptional network activity and tissue-specific signaling landscapes. Correlation analysis between ETS TF expression and immune infiltration revealed that the PEA3 subfamily members (ETV1, ETV4, and ETV5) were negatively associated with immune infiltration (Fig. 1G).

### 3.2 Melanoma Patient Clusters Classified by ETS TFs Exhibit Distinct Immune Landscapes

**Figure 2:**
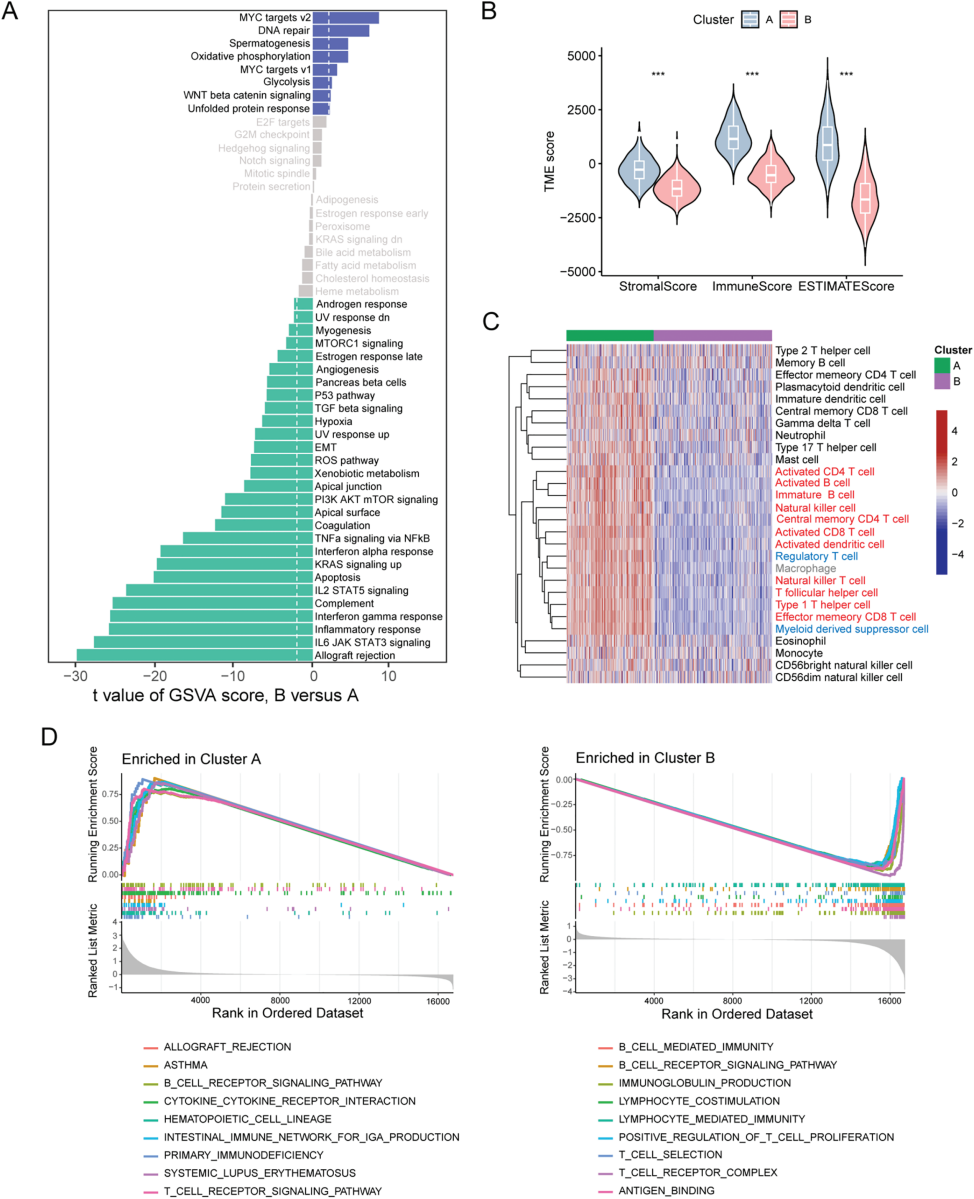
Melanoma clusters classified by ETS transcription factors exhibit distinct immune landscapes. (**A**) Differences in hallmark pathway activities scored by the GSVA algorithm between the two melanoma cluster patients. (**B**) Differences in ESTIMATE immune scores between the two melanoma patient clusters. (**C**) Heatmap of infiltration scores for 28 immune cell types, calculated by GSVA, between the two melanoma patient clusters. (**D**) Gene Ontology (GO, left panel) and KEGG (right panel) enrichment analysis based on GSEA for the two melanoma patient clusters. ***p < 0.001

We next investigated the molecular signaling differences underlying the distinct survival outcomes between the two patient clusters. Analysis of gene signatures of the hallmark pathways highlighted that immune-related signatures, such as IL6/JAK/STAT3 signaling, inflammatory response, and interferon-gamma response, were significantly enriched in Cluster A patients, while MYC targets and glycolysis, which contribute to tumor progression, were the top enriched signatures in Cluster B patients (Fig. 2A). We applied the ESTIMATE algorithm to assess immune cell infiltration within the TME. Results showed that Cluster B patients had significantly lower estimates and immune scores (Fig. 2B), suggesting lower tumor purity and reduced immune cell infiltration within the TME in this patient cluster. We examined the immune cell types that contribute to reduced immune infiltration. Analysis of 28 immune cell types revealed that the TME of Cluster B patients exhibited lower infiltration of most immune cell populations. Remarkably, among the cell types that displayed a considerable decrease in Cluster B, most were involved in active anti-tumor responses, including activated CD4^+^ T cells, activated CD8^+^ T cells, activated B cells, natural killer cells, and activated dendritic cells (Fig. 2C). This suggests a less robust anti-tumor immune response in Cluster B patients. Notably, a reduction was also observed in immunosuppressive cell types, such as MDSCs and Tregs. GSEA analyses of KEGG pathways and GO terms also revealed positive enrichment of immune processes in Cluster A patients but negative enrichment of immune functions in Cluster B patients (Fig. 2D). Together, these data suggest that melanoma patients in Cluster B, as identified by ETS TF expression, exhibit diminished immune infiltration and activity, which potentially accounts for their poor prognosis.

### 3.3 ETV4 Is Preferentially Expressed in Melanoma Cells and Affects the Crosstalk with Surrounding Immunocytes

**Figure 3:**
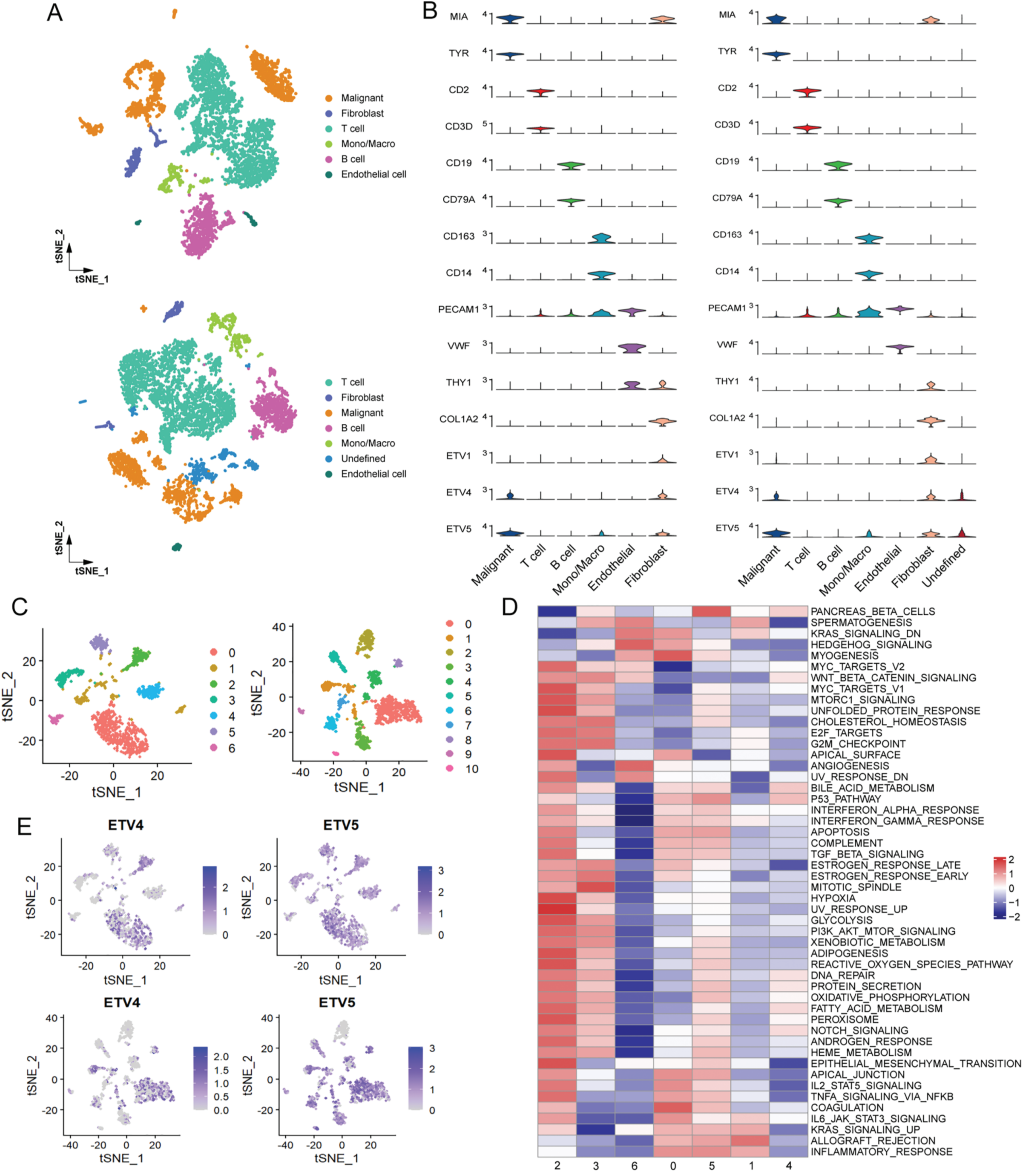
Preferential expression of ETV4 in melanoma cells. (**A**) t-SNE plots of the main cell types identified in two melanoma scRNA-seq datasets: GSE72056 (upper panel) and GSE115978 (lower panel). (**B**) Violin plots showing the expression of cell marker genes and PEA3 subfamily members (ETV1, ETV4, and ETV5) in GSE72056 (left panel) and GSE115978 (right panel). (**C**) t-SNE plots of melanoma cell subgroups in GSE72056 (left panel) and GSE115978 (right panel). (**D**) Differences in hallmark pathway activities, scored per cell using GSVA, between different melanoma cell subgroups in GSE72056. (**E**) t-SNE plot color-coded for expression of ETV4 and ETV5 in melanoma cell subgroups

To gain further insights into the melanoma TME, single-cell RNA sequencing data from two melanoma cohorts were utilized. Cells were classified into distinct groups using graph-based clustering and annotated into six primary cell lineages (Fig. 3A). Malignant cells were identified through the expression levels of the marker genes MIA and TYR. The expression of classical marker genes for each cell type was shown in Fig. 3B. Given the observed negative correlation of the expression of ETV1, ETV4, and ETV5 with immune cell infiltration, we further investigated their expression patterns across different cell lineages. Our analysis revealed that, in both cohorts, ETV1 exhibited specific expression in fibroblasts, ETV4 was primarily expressed in malignant cells and fibroblasts, while ETV5 was observed in malignant cells, fibroblasts, and also in monocytes/macrophages (Figs. 3B and S1A). Reclustering of malignant cells revealed multiple subgroups (Fig. 3C). We next attempted to identify whether heterogeneity existed among these subgroups. Analysis of hallmark pathway signatures revealed that despite the differing numbers of malignant subgroups, consistent heterogeneity was observed in these two cohorts: specific subgroups (subgroup 2 in GSE72056 and subgroup 9 in GSE115978) exhibited elevated activity in most hallmark pathways, while certain subgroups (subgroup 6 in GSE72056 and subgroup 10 in GSE115978) demonstrated reduced activity in the majority of these hallmark pathways (Figs. 3D and S1B). Notably, ETV4 expression was confined to a few malignant subgroups, whereas ETV5 was observed in a broader range of malignant subgroups (Fig. 3E). Despite the observed heterogeneity among malignant cells, the absence of detailed clinical data makes it difficult to rule out the potential influence of clinical factors, such as treatment history, on the observed intercellular heterogeneity.

**Figure 4:**
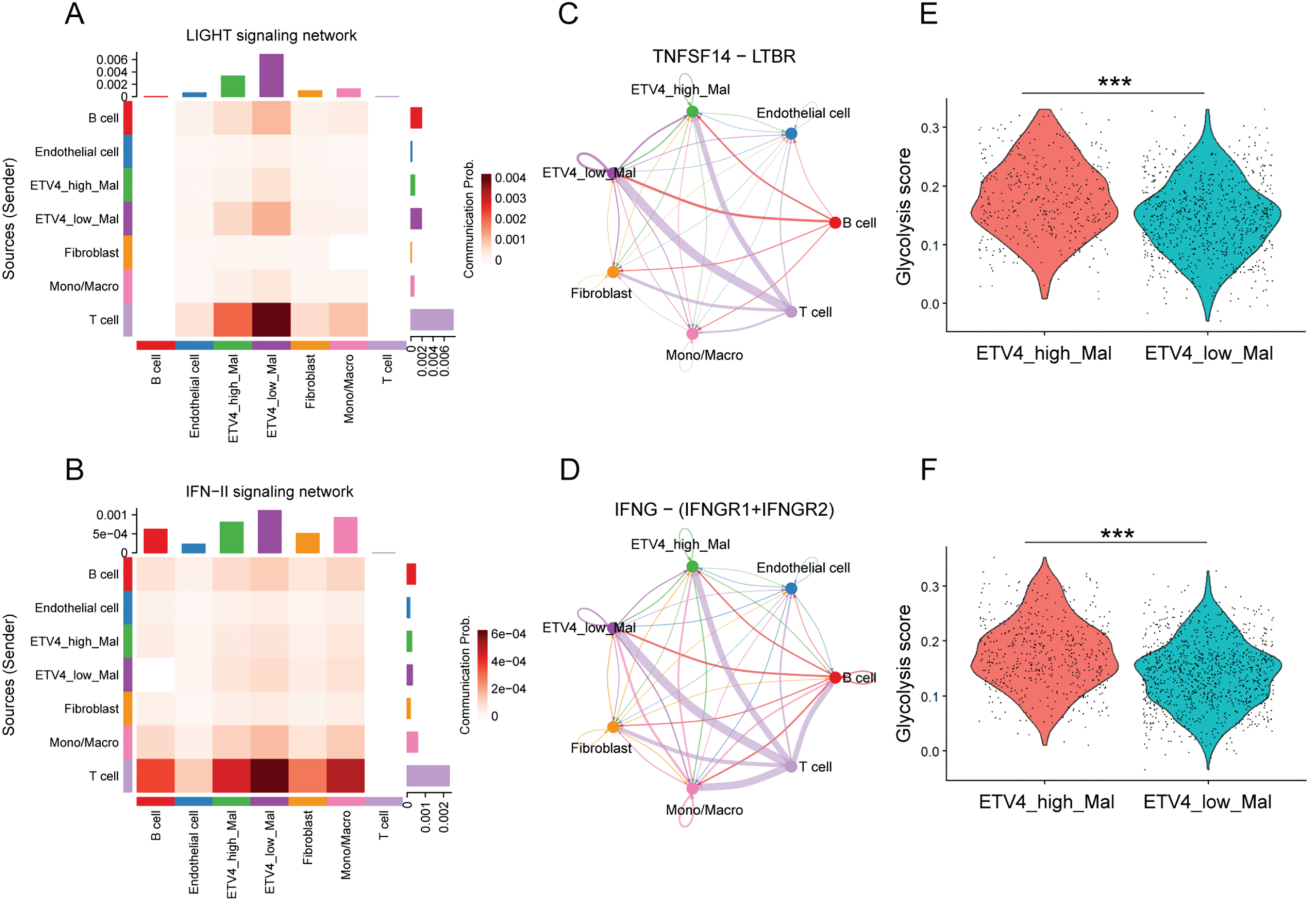
Differential cell-cell communication in ETV4-high and ETV4-low melanoma cells. (**A**,**B**) Heatmaps showing inferred communication of the LIGHT signaling network (**A**) and IFN-II signaling network (**B**) between cell types in GSE72056. (**C**) Differential communication activity of TNFSF14-LTBR in the LIGHT signaling network from T cells to ETV4-high and ETV4-low melanoma cells. Edge width represents communication probability in GSE72056. (**D**) Differential communication activity of IFNG-(IFNGR1+IFNGR2) in the IFN-II signaling network from T cells to ETV4-high and ETV4-low melanoma cells in GSE72056. (**E**,**F**) Differences in glycolysis activity, scored per cell by GSVA, between ETV4-high and ETV4-low melanoma cells in GSE72056 (**E**) and GSE115978 (**F**). ***p < 0.001

We asked whether differing expression levels of ETV4 in malignant cells influence communications with surrounding immunocytes. Malignant melanoma cells were classified into ETV4-high and ETV4-low groups based on the upper tertile of ETV4 expression. The overall landscape of intercellular communication among cell lineages in the melanoma TME was evaluated by analyzing ligand-receptor interactions. Notably, ETV4-low malignant cells demonstrated stronger interaction networks with T cells compared to their ETV4-high counterparts in both cohorts (Fig. S2A). A more detailed examination of the crosstalk between malignant cells and T cells revealed diminished LIGHT (also known as TNFSF14) and IFN-II signaling in ETV4-high malignant cells relative to ETV4-low malignant cells (Figs. 4A,B and S2B). Specifically, the TNFSF14-LTBR and IFNG-(IFNGR1+IFNGR2) ligand-receptor pairs were identified as the primary mediators responsible for the decreased LIGHT and IFN-II signaling in ETV4-high malignant cells during their interactions with T cells (Figs. 4C,D and S2C). Both TNFSF14 and IFNG, through their respective receptors LTBR and IFNGR1/IFNGR2, play crucial roles in antitumor immunity by promoting both innate and adaptive immune responses [25–27]. Moreover, GSVA analysis of the glycolysis signature, a cancer hallmark associated with T cell anergy and immune escape [28,29], at the single-cell level revealed that ETV4-high malignant cells exhibited significantly higher glycolysis activity (Fig. 4E,F), which is consistent with our previous findings in breast cancer [30]. Based on the above findings, we hypothesized that ETV4 expression in melanoma cells was significantly involved in their tolerance to immune attack by T cells.

### 3.4 Downregulation of ETV4 Enhances T Cell-Mediated Tumor Killing and Suppresses Tumor Growth in Immunocompetent Mice

**Figure 5:**
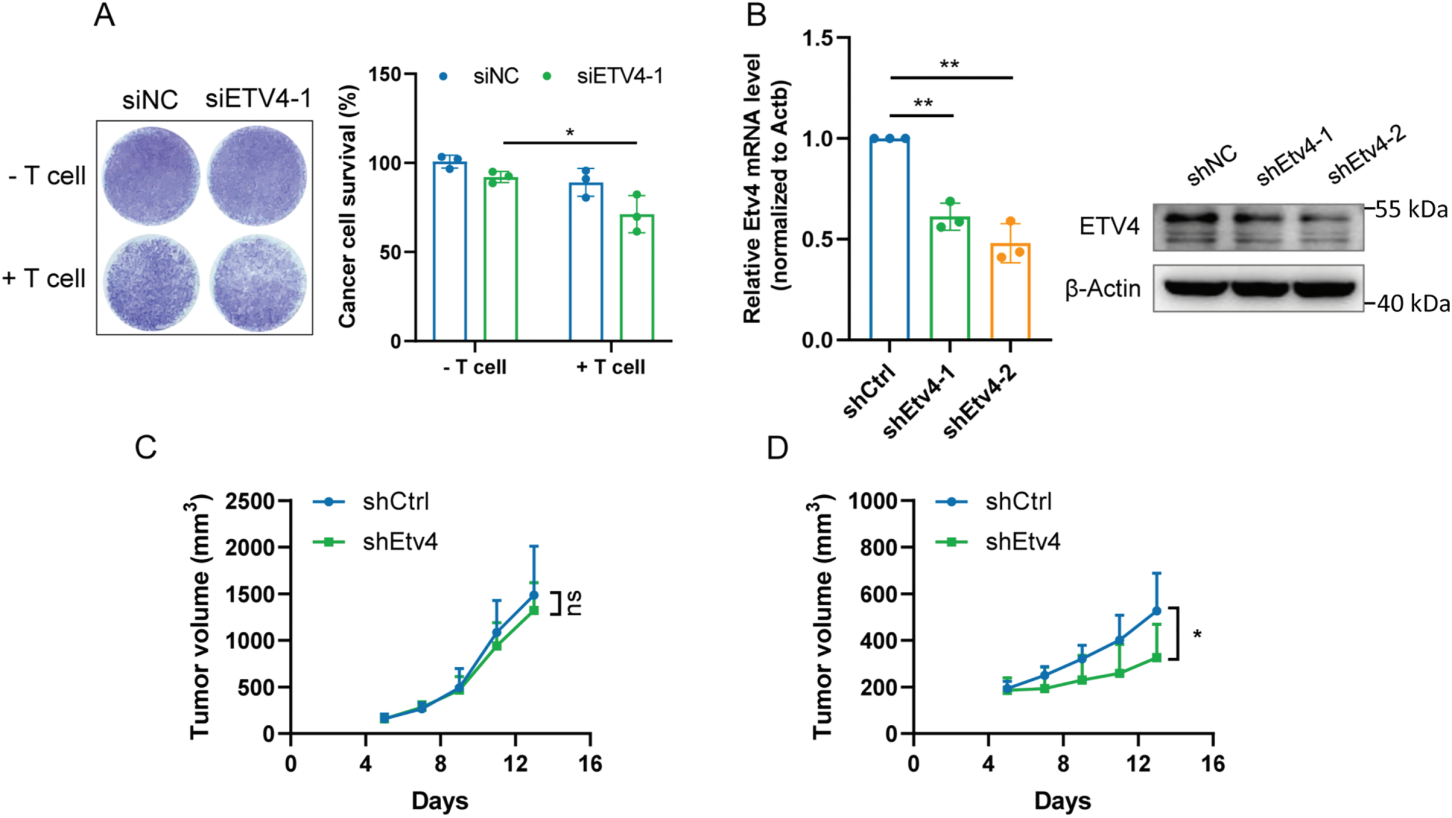
ETV4 knockdown enhances T cell-mediated tumor killing and suppresses tumor growth in immunocompetent mice. (**A**) Results of T cell-mediated cancer cell-killing assay. Representative images of crystal violet-stained SK-MEL-28 melanoma cells co-cultured with or without T cells (left) and quantification of cancer cell survival (right). (**B**) Etv4 expression in MC38 cells with stable Etv4 knockdown was measured by qRT-PCR (left) and immunoblotting (right). (**C**,**D**) MC38 tumor growth in immunodeficient BALB/c nude mice (**C**) and in immunocompetent C57BL/6 mice (**D**). ns, not significant; *p < 0.05, **p < 0.01

To test whether ETV4 expression contributes to tumor resistance to T cell killing, we extracted T cells from human peripheral blood and cocultured them with SK-MEL-28 melanoma cells. We observed that ETV4 downregulation in melanoma cells rendered them more sensitive to T cell-mediated killing (Fig. 5A). To assess the effect of tumor ETV4 expression on *in vivo* antitumor immune activity, murine MC38 cells with stable downregulation of Etv4 were constructed (Fig. 5B). We inoculated shEtv4 MC38 cells and their control counterparts into immunocompetent C57BL/6 mice and immunodeficient BALB/c nude mice. We found that while there was no significant difference in tumor growth between the Etv4 knockdown and control groups in immunodeficient mice, Etv4 knockdown in immunocompetent mice significantly suppressed MC38 tumor growth (Fig. 5C,D). This suggests that Etv4 downregulation benefits the immune system’s anti-tumor response.

### 3.5 ETV4 Acts as a Transcriptional Activator of PD-L1 Expression in Melanoma Cells

**Figure 6:**
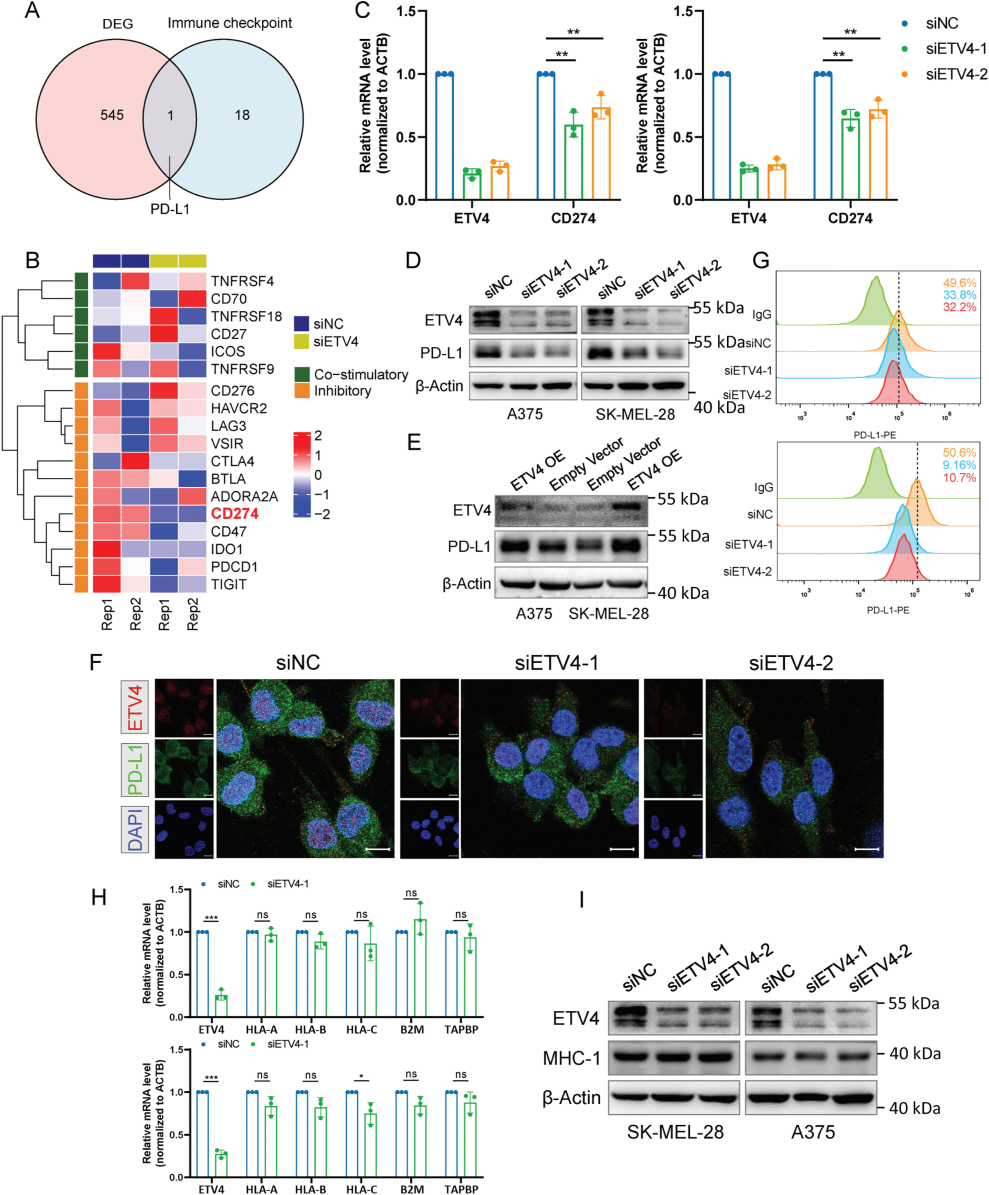
Identification of PD-L1 as a downstream target of ETV4 in melanoma cells. (**A**) Venn diagram showing PD-L1 as the sole common gene from the intersection of DEGs between ETV4 knockdown and control SK-MEL-28 cells with immune checkpoints. (**B**) Heatmap of RNA-seq FPKM data for immune checkpoint genes in SK-MEL-28 cells. (**C**) PD-L1 mRNA expression measured by qRT-PCR in A375 (left) and SK-MEL-28 (right) cells after ETV4 knockdown. (**D**,**E**) PD-L1 protein expression measured by immunoblotting in A375 and SK-MEL-28 cells after ETV4 knockdown (D) and ETV4 overexpression (**E**). (**F**) Immunofluorescence microscopy images of ETV4 (red), PD-L1 (green), and DAPI (blue) in SK-MEL-28 cells with ETV4 knockdown. Scale bars, 10 μm. (**G**) Flow cytometry analysis of surface PD-L1 expression in A375 (upper) and SK-MEL-28 (lower) cells after ETV4 knockdown. (**H**) qRT-PCR analysis of MHC-I-related gene (HLA-A, HLA-B, B2M, and TAPBP) mRNA expression in A375 (upper) and SK-MEL-28 (lower) cells after ETV4 knockdown. (**I**) MHC-I complex expression measured by immunoblotting after ETV4 knockdown. ns, not significant; *p < 0.05; **p < 0.01; ***p < 0.001

After identifying that ETV4 is negatively associated with the tumor-killing activity of T cells, we proceeded to investigate the underlying mechanism. We hypothesized that ETV4 may regulate the expression of immune checkpoint(s). SK-MEL-28 cells with ETV4 knockdown were subjected to RNA sequencing. Differential gene expression analysis identified 261 up-regulated and 285 down-regulated genes upon ETV4 knockdown (Fig. S3A). Motif enrichment analysis was performed using HOMER on the promoter regions of these 546 DEGs. The analysis identified several significantly enriched motifs, indicating that ETV4 may exert its regulatory functions by directly or indirectly influencing specific cis-regulatory elements. The top 10 enriched motifs are shown in Supplementary Table S4. These DEGs were then cross-referenced with a previously reported set of immune checkpoint genes [31]. Notably, this analysis pinpointed PD-L1 as the sole gene of interest (Fig. 6A), underscoring its relevance in this context. The fragments per kilobase million (FPKM) expression data for immune checkpoint genes are shown in Fig. 6B. The downregulation of PD-L1 mRNA upon ETV4 knockdown was further validated in A375 and SK-MEL-28 cells (Fig. 6C). Additionally, a marked reduction in PD-L1 protein levels was observed in the ETV4 knockdown groups compared to the control (Figs. 6D and S3B), while ectopic expression of ETV4 resulted in a significant upregulation of PD-L1 protein levels (Fig. 6E). Similar results were consistently observed in the immunofluorescence assay performed on SK-MEL-28 cells with ETV4 downregulation (Fig. 6F). The surface expression of PD-L1 is critical for its interaction with the PD-1 receptor on T cells and inhibition of T cell activation. Flow cytometry analysis revealed a significant decrease in surface PD-L1 expression following ETV4 knockdown (Fig. 6G). Collectively, these results suggest that ETV4 expression impairs T cell-mediated tumor killing by downregulating PD-L1 at the transcriptional level, thereby contributing to immune evasion.

Dysregulated MHC-I-mediated antigen presentation is another key mechanism by which tumors evade immune surveillance. We next investigated whether ETV4 could regulate the expression of MHC-I-related genes. Results from A375 and SK-MEL-28 cells demonstrated no significant changes in the mRNA expression levels of MHC-I-related genes (HLA-A, HLA-B, B2M, and TAPBP) following ETV4 knockdown (Fig. 6H). Similarly, ETV4 downregulation did not result in significant alterations in the protein expression levels of the MHC-I complex (Fig. 6I). These findings preliminarily exclude the possibility that ETV4 contributes to tumor immune evasion via MHC-I antigen presentation.

**Figure 7:**
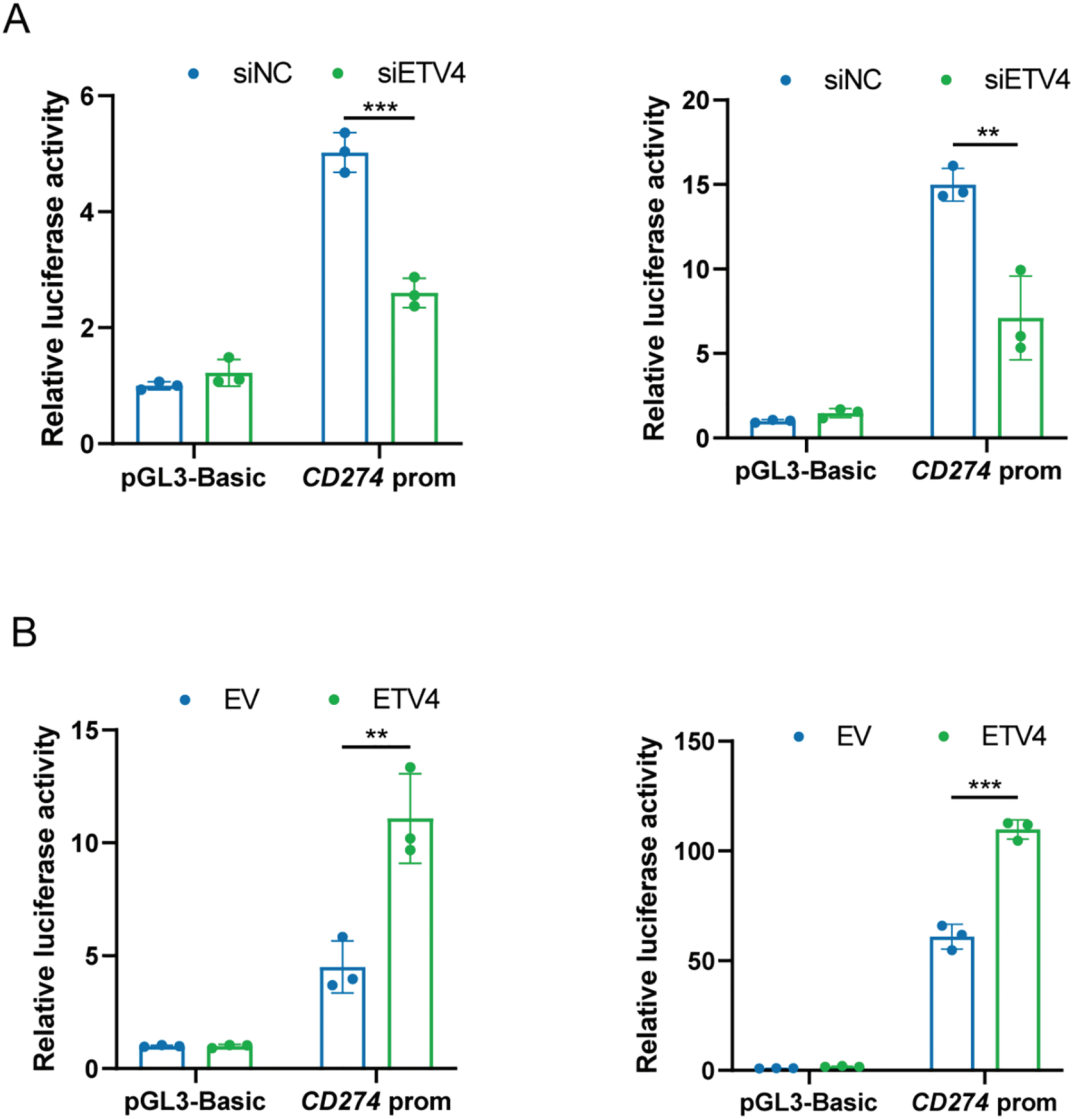
ETV4 activates *CD274* transcription in melanoma cells. (**A**) Decreased luciferase activity of the *CD274* promoter reporter in A375 (left) and SK-MEL-28 (right) cells after ETV4 knockdown. (**B**) Increased luciferase activity of the CD274 promoter reporter in A375 (left) and SK-MEL-28 (right) cells after ETV4 overexpression. **p < 0.01; ***p < 0.001

To further investigate whether ETV4, as a transcription factor, regulates PD-L1 by modulating its promoter activity, a reporter containing the putative promoter region of *CD274*, the gene encoding PD-L1, was constructed. Subsequently, a dual-luciferase reporter assay was performed. In both A375 and SK-MEL-28 cells, downregulation of ETV4 significantly reduced *CD274* promoter reporter activity, while ETV4 overexpression led to an increase in *CD274* promoter activity (Fig. 7A,B). Thus, our findings provide evidence that ETV4 is a key transcription factor promoting *CD274* gene transcription in melanoma cells.

### 3.6 Pretreatment ETV4 Levels Were Correlated with Anti-PD-1 Therapy Efficacy in Melanoma Patients

**Figure 8:**
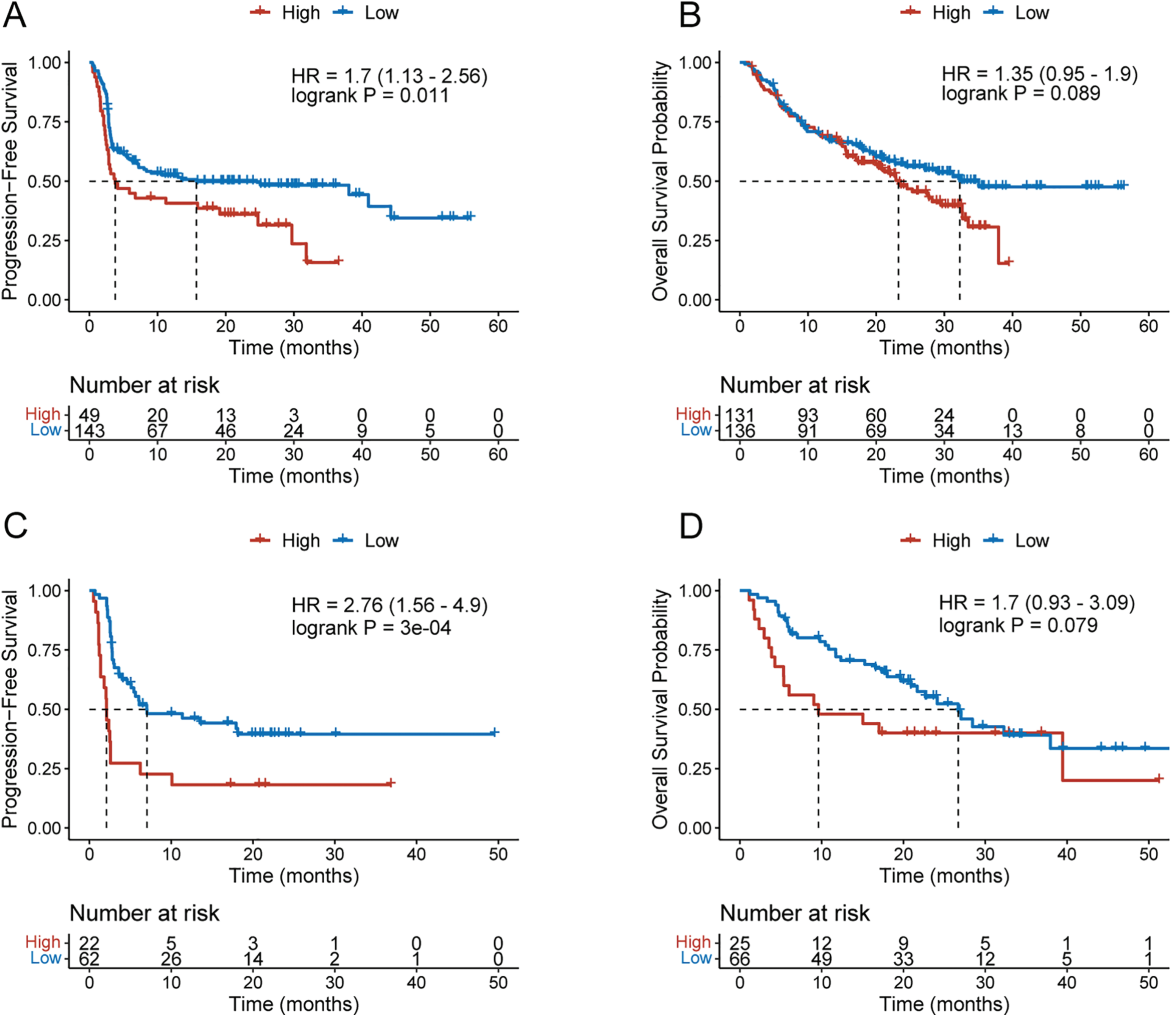
Pretreatment ETV4 levels predict anti-PD-1 efficacy in melanoma patients. (**A**,**B**) KM curves for PFS (**A**) and overall survival (**B**) in melanoma patients with high or low pretreatment ETV4 levels receiving anti-PD-1 therapy. (**C**,**D**) KM curves for PFS (**C**) and overall survival (**D**) in melanoma patients with high or low pretreatment ETV4 levels receiving anti-CTLA-4 therapy

To further assess whether ETV4 levels influence immunotherapy efficacy in melanoma patients, we analyzed melanoma cohorts with available survival data and categorized data on ETV4 expression. In patients treated with anti-PD-1 therapy, elevated ETV4 levels before treatment ETV4 levels showed a significant correlation with shorter progression-free survival (PFS) (HR = 1.7, log-rank *p* = 0.01, Fig. 8A). Additionally, while high pretreatment ETV4 levels demonstrated a trend toward poorer overall survival, the difference was statistically non-significant (log-rank *p* = 0.089), indicating potential differences in survival outcomes that merit further investigation (Fig. 8B). Similar findings were observed in patients receiving anti-CTLA-4 therapy: high pretreatment ETV4 levels were strongly correlated with reduced PFS (HR = 2.76, log-rank *p* < 0.001, Fig. 8C); and although a trend toward worse overall survival was observed in these patients, the difference failed to reach the threshold of statistical significance (*p* = 0.079, Fig. 8D), which may be related to the limited sample size. These findings suggest that elevated pretreatment ETV4 levels act a predicor for poor PFS in melanoma patients undergoing anti-PD-1 or anti-CTLA-4 therapy.

## 4 Discussion

Despite the breakthrough advances of ICIs in cancer therapy, their broader clinical application remains hindered by several challenges, such as limited efficacy in majority of cancer patients, the development of acquired resistance, and the absence of general robust predictive biomarkers [32,33]. The low response rates to immunotherapy are largely attributed to the immunosuppressive state of the TME, where transcriptional regulation plays a critical role in shaping the immune landscape [34]. A recent study reported that TF-based profiling underlies the classification of epigenetic and transcriptomic subtypes of colorectal cancer [23], highlighting the potential of transcription factor programs in defining cancer subtypes. In this study, based on the expression patterns of the ETS TF family, which is associated with cancer progression [13], we identified two cancer subtypes (Cluster A and Cluster B) in both melanoma and hepatocellular carcinoma, representing immunologically “hot” and “cold” tumor types, respectively. Cluster A and Cluster B showed significantly different overall survival outcomes, indicating the potential of ETS transcription factor expression as a prognostic indicator. Moreover, we demonstrated that tumor ETV4 expression influences the crosstalk with infiltrating immune cells within the melanoma TME and affects the response to anti-PD-1 therapy, potentially due to its role in transcriptionally activating CD274 expression.

Multiple ETS transcription factors were found to be expressed in melanoma and they exhibited redundant functions [35]. Although targeting multiple ETS family members has been recognized as a therapeutic strategy showing efficacy in impeding melanoma progression, their roles in regulating the anti-tumor immune response in melanoma remain unclear. Activation of the immune microenvironment is a prerequisite for benefiting from immune checkpoint blockade therapies [36]. Cluster B melanomas demonstrated reduced infiltration of immune cells and lower enrichment of immune-related pathway activity. In Cluster B melanomas, which exhibited worse prognosis, the expression levels of ETV2, ERF, ELK1, ETV4, and ETV5 were higher compared to those in Cluster A. Therefore, our study suggests that the expression patterns of the ETS family members reflect the immune landscape and are involved in regulating anti-tumor immune activity within the melanoma TME.

Given that ETS family members are also expressed in immune cells, such as ETS1 in T cells [14] and ELK1 in B cells [37], we utilized scRNA-seq data to analyze the expression of the PEA3 subfamily, which was found to be associated with immune infiltration, in melanoma cells. Our study revealed a preferential and variable expression of ETV4 in melanoma cells, which exhibit heterogeneity in hallmark pathway activity. Melanoma cells can shape the local immune landscape by recruiting pro-tumor immune subsets, such as myeloid-derived suppressor cells, while suppressing or excluding anti-tumor immune cells, such as effector T cells [38]. These processes are largely mediated through the secretion of various cytokines and chemokines by melanoma cells. Our ligand-receptor-based cell communication analysis demonstrated increased interactions between ETV4-low melanoma cells and T cells, including TNFSF14-LTBR and IFNG-(IFNGR1+IFNGR2) signaling pathways. Elevated activity of these two representative pathways augments the function of effector T cells, leading to a more robust anti-tumor immune response [39,40]. While a recent study has shown that ETV4 plays a role in melanoma cell proliferation and migration [41], our findings suggest a novel role for tumor ETV4 expression in melanoma progression through the regulation of tumor-immune cell interactions.

Immune checkpoint inhibitors target the dysfunctional immune system to promote elimination of tumor cells by effector T cells [42]. We demonstrated that pretreatment levels of ETV4 are associated with a reduced sensitivity to anti-PD-1 therapy. Immune checkpoint signaling modulates T cell function, and our RNA-seq analysis of SK-MEL-28 cells identified PD-L1 as the predominant immune checkpoint regulated by ETV4 in melanoma, consistent with findings in hepatocellular carcinoma [16]. Coculture of human CD8^+^ T cells with SK-MEL-28 cells under direct physical contact demonstrated that ETV4 downregulation enhances the *in vitro* tumor-killing ability of CD8^+^ T cells. Regulation of PD-L1 is a key mechanism that significantly influences the efficacy of ICIs. Our study suggests that the association between pretreatment levels of ETV4 and the efficacy of anti-PD-1/anti-CTLA-4 therapy may be explained by the positive regulation of PD-L1 expression by ETV4 in melanoma.

Transcription factors modulate immunotherapy efficacy through diverse mechanisms. For example, STAT3 enhances immune suppression by upregulating PD-L1, inhibiting dendritic cell maturation, and inducing M2 macrophage polarization with IL-10 and TGF-β production [43–45]. Similarly, HIF-1α, a hypoxia-inducible factor stabilized under low oxygen conditions, contributes to immunotherapy resistance by upregulating PD-L1, promoting a metabolic shift toward aerobic glycolysis, and mediating metabolic reprogramming of CD8^+^ T cells [46,47]. In this study, we found that ETV4 upregulates PD-L1 transcription in melanoma, consistent with prior findings in liver cancer where ETV4 binds the CD274 promoter, with the −734 to −339 bp region being essential for transcriptional regulation [16]. Our previous work also showed that ETV4 regulates key glycolytic enzymes, including HK2 and LDHA [30], suggesting that ETV4 and HIF-1α may act synergistically in metabolic reprogramming that contributes to immunotherapy resistance. Additionally, ETV4 regulates the expression of cytokines and cytokine receptors such as CCL2 and CXCR4, promoting tumor-associated macrophages (TAMs) and MDSCs recruitment and fostering an immunosuppressive microenvironment [16,30]. The dual role in both tumor metabolism and immune cell recruitment may distinguish ETV4 from other transcription factors associated with immunotherapy resistance.

Despite our findings that targeting ETV4 may potentiate anti-tumor immunity and improve responses to immune checkpoint blockade in melanoma, translating ETV4-targeted therapies into clinical practice presents a significant challenge. Transcription factors are traditionally considered “undruggable”. Currently, there are no approved small-molecule inhibitors or proteolysis targeting chimera (PROTAC) degraders that specifically target ETV4. However, recent advances in targeted protein degradation technologies, including PROTACs and molecular glues, have demonstrated promise in eliminating difficult-to-target transcription factors such as STAT3 and MYC [48–50]. These approaches may present as viable strategies for targeting ETV4. Alternatively, in the absence of direct inhibitors, indirect targeting of downstream immune modulators represents a more feasible near-term approach. For example, targeting metabolic enzymes or cytokine signaling pathways regulated by ETV4 could serve as alternative routes to reprogram the tumor immune microenvironment. Additionally, emerging computational drug repurposing strategies have shown promise in identifying approved compounds that may modulate transcription factor-associated pathways indirectly, providing a potential framework for identifying candidate agents that may regulate ETV4-associated networks [51].

Furthermore, our scRNA-seq analysis revealed that ETV4 was detected in cancer-associated fibroblasts (CAFs), a finding that points to the potential function of ETV4 in stromal remodeling and stromal-immune-tumor interactions. Prior studies have demonstrated that CAFs produce secreted factors, exosomes and metabolites that influence various tumor phenotypes such as angiogenesis, immunology and metabolism [52,53]. While our study primarily focused on tumor cell-intrinsic ETV4, its expression in stromal components raises the possibility of off-target effects in future systemic targeting strategies. This complexity highlights the need for cell type-specific modulation approaches and warrants further investigation.

There are several limitations in this study. First, although we identified two distinct clusters of patients with significantly different prognoses according to the expression patterns of ETS TFs in both melanoma and hepatocellular carcinoma, we have not yet validated these findings with our own patient samples. Future research will prioritize the collection of additional samples for validation purposes. Second, in our analysis of signaling communication between melanoma cells and T cells, we did not perform further identification of T cell subpopulations due to the limited number of cells. As different T cell subpopulations have distinct immunological functions, ETV4 expression in tumors may differentially affect these subsets. Additionally, we did not experimentally validate whether PD-L1 is the sole downstream mediator of ETV4-induced immune evasion in melanoma. Further functional studies, such as rescue experiments combining ETV4 knockdown with PD-L1 overexpression, are warranted. Finally, in our animal experiments, we did not assess the changes in immune cell infiltration within the TME following ETV4 downregulation. Future studies incorporating immune profiling *in vivo* will help further elucidate the immunomodulatory role of ETV4.

In summary, our study reveals that the expression patterns of the 28 ETS family members can be used to dichotomize melanoma into two clusters with differing overall survival outcomes and distinct immune infiltration landscapes. ETV4 expression in melanoma cells influences their crosstalk with T cells and impairs T cell-mediated tumor killing by upregulating PD-L1 expression. High pretreatment levels of ETV4 are indicative of low responsiveness to immune checkpoint inhibitors in melanoma patients. Our study provides theoretical support for targeting ETV4 as a potential therapeutic strategy, particularly in combination with ICIs, which may result in enhanced therapeutic efficacy.

## Supplementary Materials

Figure S1 Single-cell transcriptomic characterization of marker genes, PEA3 subfamily expression, and pathway activities in melanoma. (A) Dot plot showing the expression levels of marker genes and PEA3 subfamily members in different cell types in GSE72056 (left) and GSE115978 (right). (B) Differences in hallmark pathway activities, scored per cell using GSVA, between different melanoma cell subgroups in GSE115978.

Figure S2 Cell–cell communication patterns and signaling network activities in melanoma. (A) Inferred overall numbers of cell-cell interactions and interaction strengths between cell types in melanoma. (B) Heatmaps showing inferred communication within the LIGHT and IFN-II signaling networks across cell types in GSE115978. (C) Differential communication activities of TNFSF14-LTBR in the LIGHT signaling network and IFNG-(IFNGR1+IFNGR2) in the IFN-II signaling network from T cells to ETV4-high and ETV4-low melanoma cells in GSE115978. Edge width represents communication probability.

Figure S3 Effects of ETV4 knockdown on transcriptomic profiles and PD-L1 expression. (A) Number of differentially expressed genes (left) and a volcano plot of differentially expressed transcripts (right) in SK-MEL-28 cells following ETV4 knockdown, as identified by RNA-seq. The thresholds for differentially expressed genes were |log2 (fold change) | > 1 and false discovery rate (FDR) < 0.05. (B) PD-L1 protein expression levels assessed by immunoblotting in MC38 and SW620 colorectal cancer cells after ETV4 knockdown.

## Data Availability

The datasets used in this study can be found in TCGA (https://portal.gdc.cancer.gov/), UCSC Xena (https://xena.ucsc.edu/), TISCH database (http://tisch.comp-genomics.org/), and Kaplan-Meier Plotter (https://kmplot.com/analysis/, accessed on 01 September 2025). The raw sequencing data for ETV4 knockdown SK-MEL-28 cells are available from the corresponding authors upon reasonable request.

## Supplementary Materials

The supplementary material is available online at https://www.techscience.com/doi/10.32604/or.2025.070180/s1.
