# Supplementary figures and images for: ETV4-Mediated PD-L1 Upregulation Promotes Immune Evasion and Predicts Poor Immunotherapy Response in Melanoma
Source: Oncol Res. 2025 Dec 30;34(1):25. doi: 10.32604/or.2025.070180 (PMC12774552; doi:10.32604/or.2025.070180)

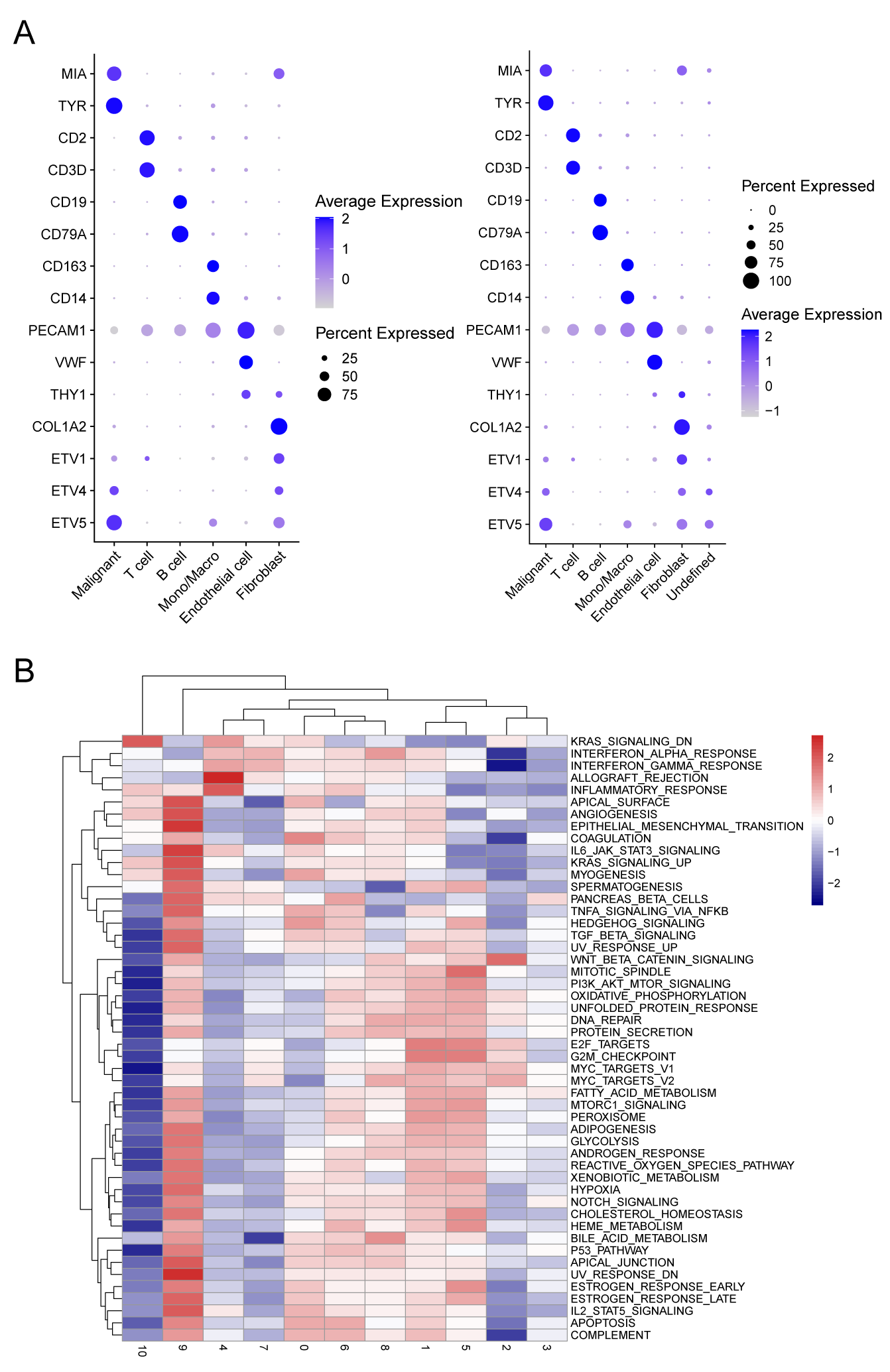

Supplement: Figure S1 [file OncolRes-34-70180-s001.tif]

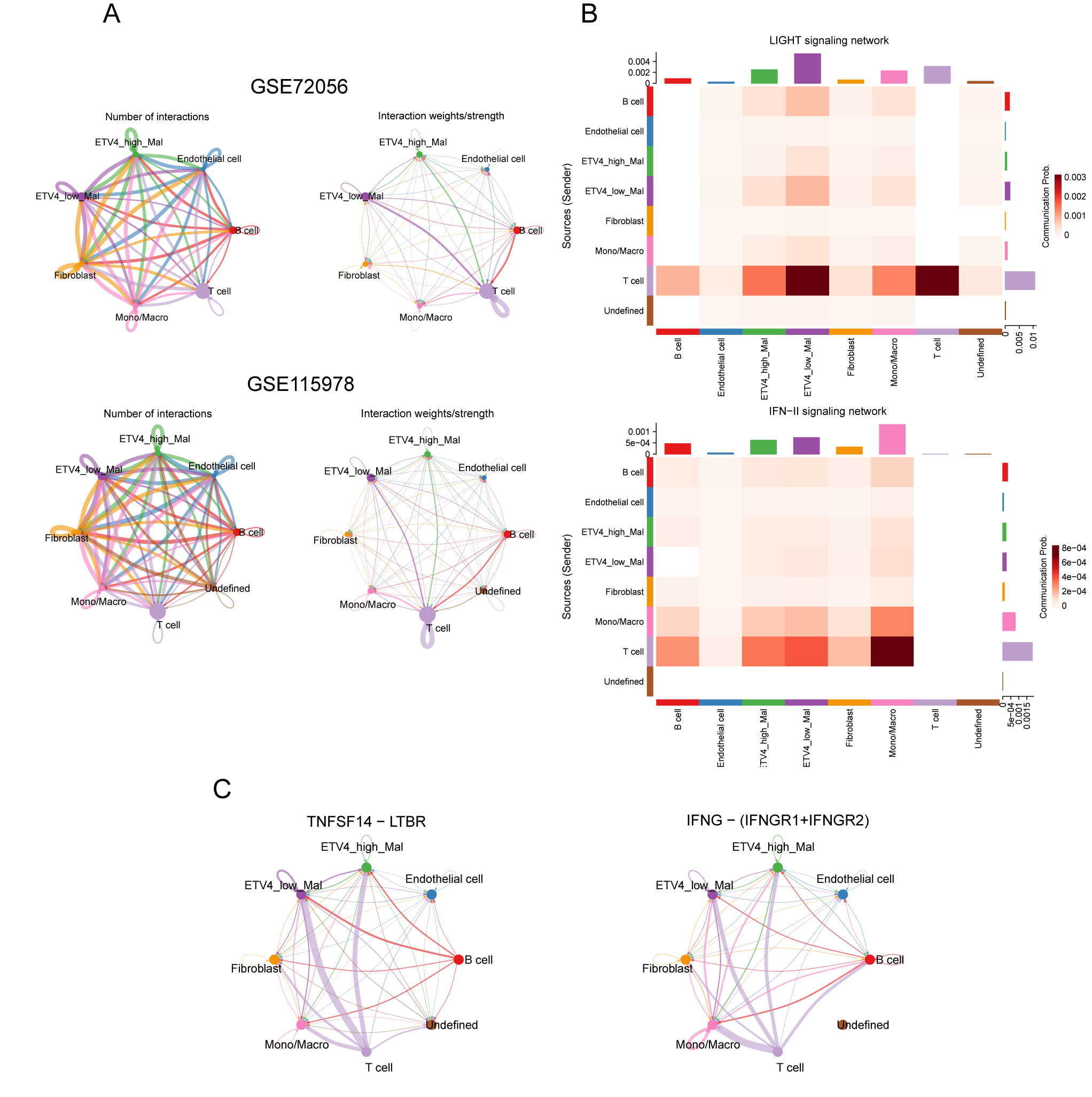

Supplement: Figure S2 [file OncolRes-34-70180-s002.tif]

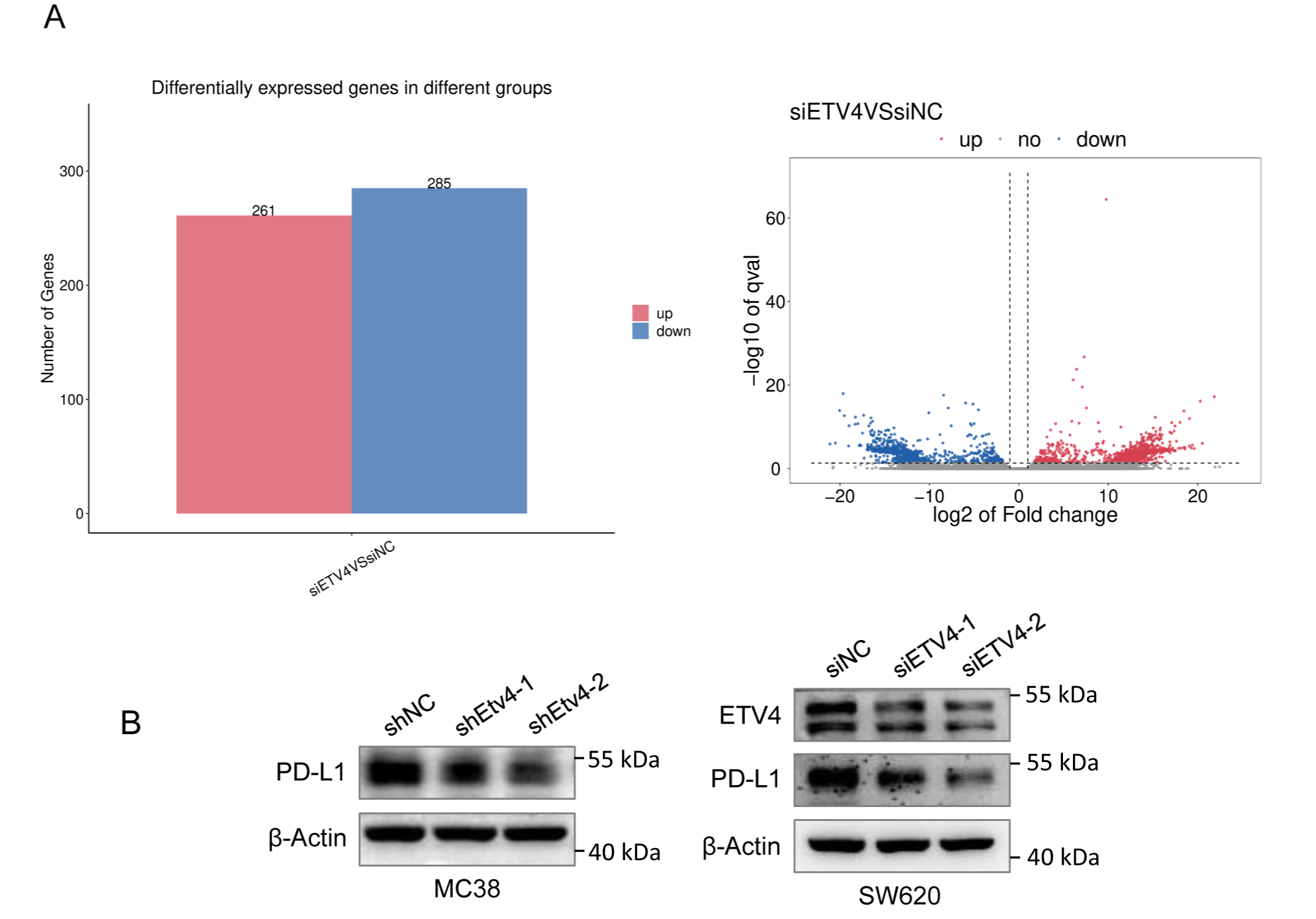

Supplement: Figure S3 [file OncolRes-34-70180-s003.tif]
