# Supplementary material for: ETV4-Mediated PD-L1 Upregulation Promotes Immune Evasion and Predicts Poor Immunotherapy Response in Melanoma
Source: Oncol Res. 2025 Dec 30;34(1):25. doi: 10.32604/or.2025.070180 (PMC12774552; doi:10.32604/or.2025.070180)
Supplement: Supplementary file 4 [file OncolRes-34-70180-s004.docx]

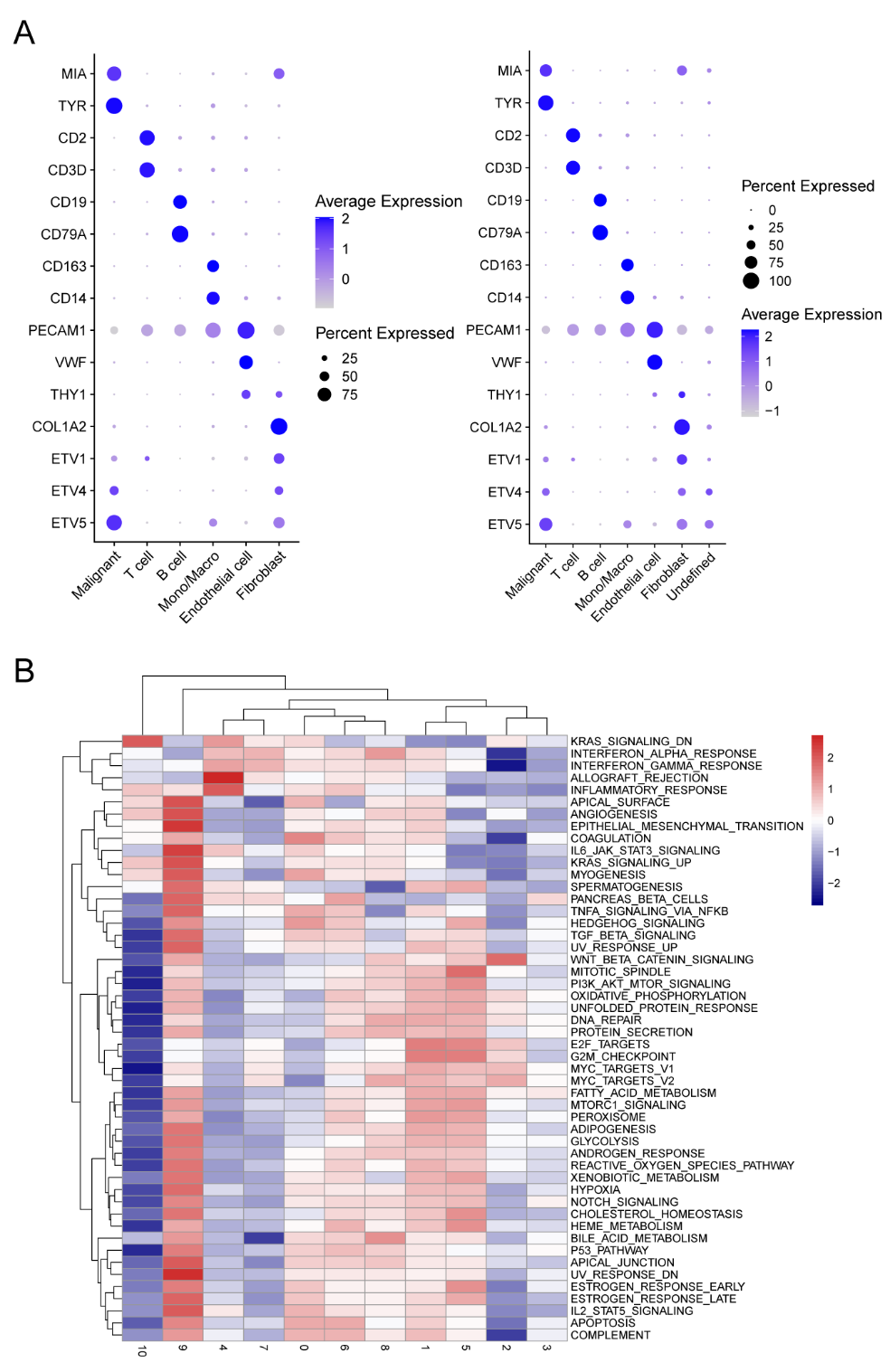


Figure S1. Single-cell transcriptomic characterization of marker genes, PEA3 subfamily expression, and pathway activities in melanoma. (A) Dot plot showing the expression levels of marker genes and PEA3 subfamily members in different cell types in GSE72056 (left) and GSE115978 (right). (B) Differences in hallmark pathway activities, scored per cell using GSVA, between different melanoma cell subgroups in GSE115978.


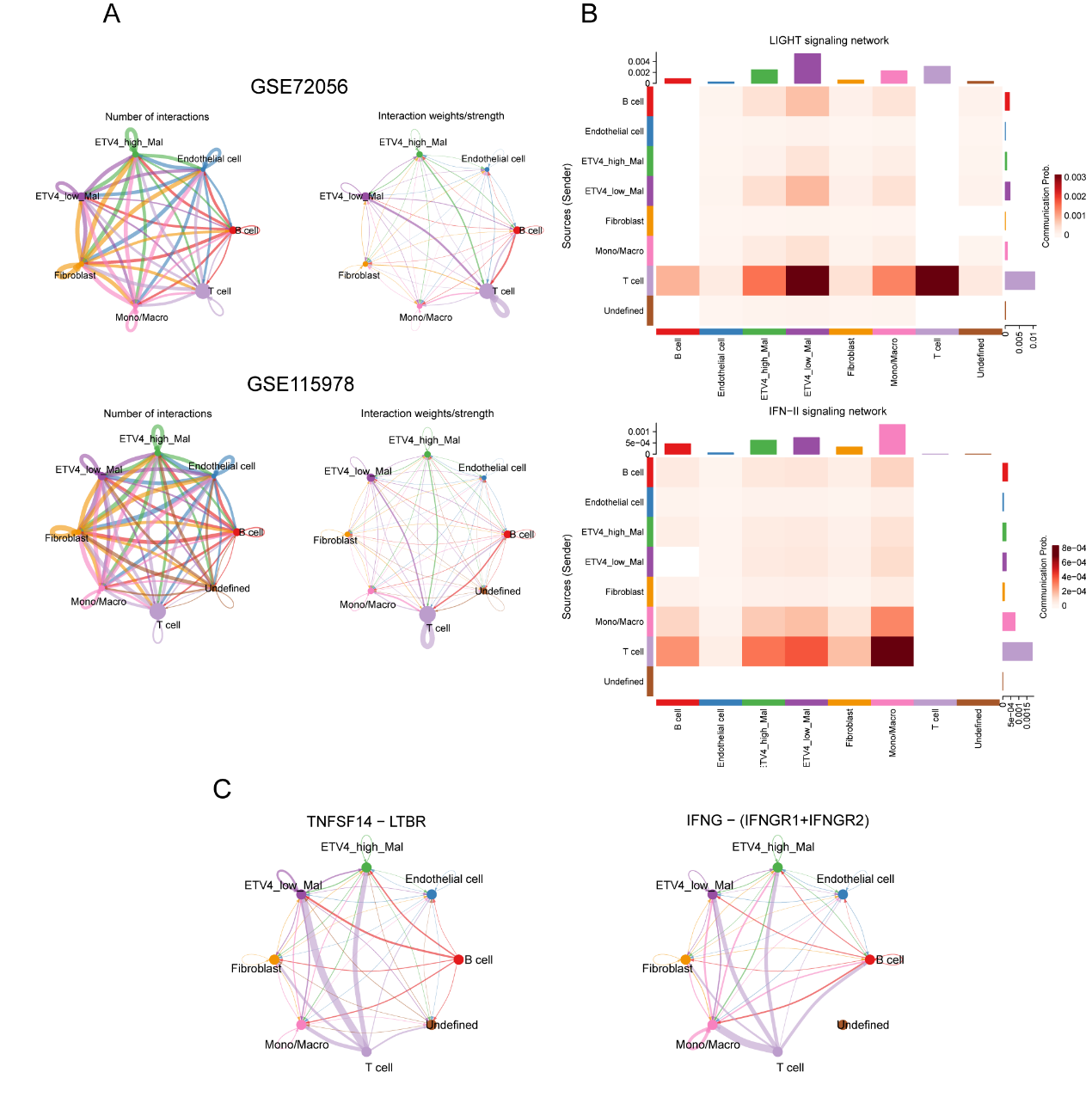


Figure S2. Cell–cell communication patterns and signaling network activities in melanoma. (A) Inferred overall numbers of cell-cell interactions and interaction strengths between cell types in melanoma. (B) Heatmaps showing inferred communication within the LIGHT and IFN-II signaling networks across cell types in GSE115978. (C) Differential communication activities of TNFSF14-LTBR in the LIGHT signaling network and IFNG-(IFNGR1+IFNGR2) in the IFN-II signaling network from T cells to ETV4-high and ETV4-low melanoma cells in GSE115978. Edge width represents communication probability.


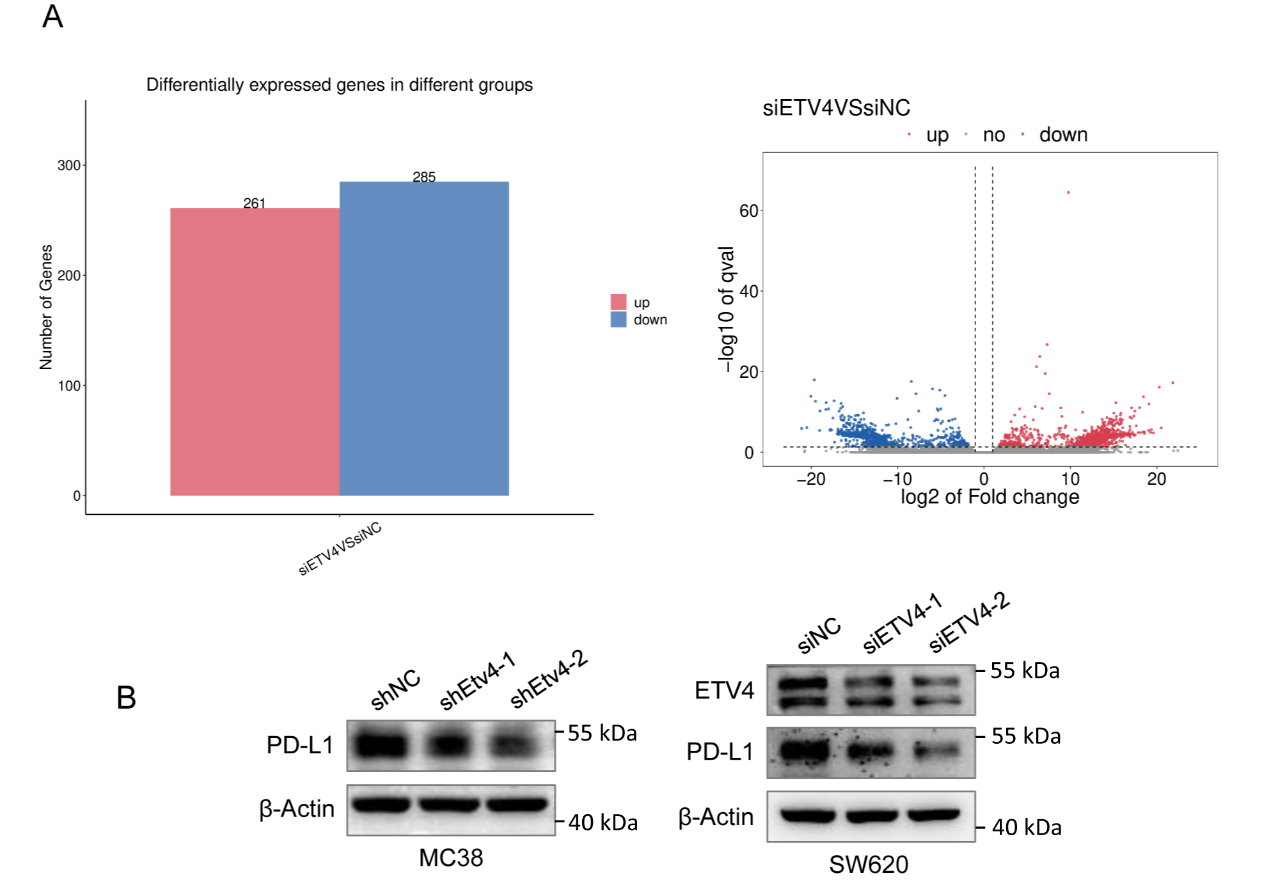


Figure S3. Effects of ETV4 knockdown on transcriptomic profiles and PD-L1 expression. (A) Number of differentially expressed genes (left) and a volcano plot of differentially expressed transcripts (right) in SK-MEL-28 cells following ETV4 knockdown, as identified by RNA-seq. The thresholds for differentially expressed genes were |log2 (fold change) | > 1 and false discovery rate (FDR) < 0.05. (B) PD-L1 protein expression levels assessed by immunoblotting in MC38 and SW620 colorectal cancer cells after ETV4 knockdown.
