# Supplementary material for: ETV4-Mediated PD-L1 Upregulation Promotes Immune Evasion and Predicts Poor Immunotherapy Response in Melanoma
Source: Oncol Res. 2025 Dec 30;34(1):25. doi: 10.32604/or.2025.070180 (PMC12774552; doi:10.32604/or.2025.070180)
Supplement: Supplementary file 5 [file OncolRes-34-70180-s005.docx]

Table S2. Knockdown shRNA sequences used in this study

| Target Gene | Sequence |
| --- | --- |
| shEtv4-1 | GATCTCGGCCACAGAGGTGGATATTCTCGAGAATATCCACCTCTGTGGCCGATTTTTG |
| shEtv4-2 | GATCCCCAACAAATGCTCATTTCATCTCGAGATGAAATGAGCATTTGTTGGGTTTTTG |
