# Supplementary material for: ETV4-Mediated PD-L1 Upregulation Promotes Immune Evasion and Predicts Poor Immunotherapy Response in Melanoma
Source: Oncol Res. 2025 Dec 30;34(1):25. doi: 10.32604/or.2025.070180 (PMC12774552; doi:10.32604/or.2025.070180)
Supplement: Supplementary file 6 [file OncolRes-34-70180-s006.docx]

Supplementary Table 4. Top 10 enriched motifs identified by HOMER analysis of promoters of differentially expressed genes after ETV4 knockdown

| Rank | Motif | P value | % of Targets | % of Background | STD (Bg STD) |
| --- | --- | --- | --- | --- | --- |
| 1 | 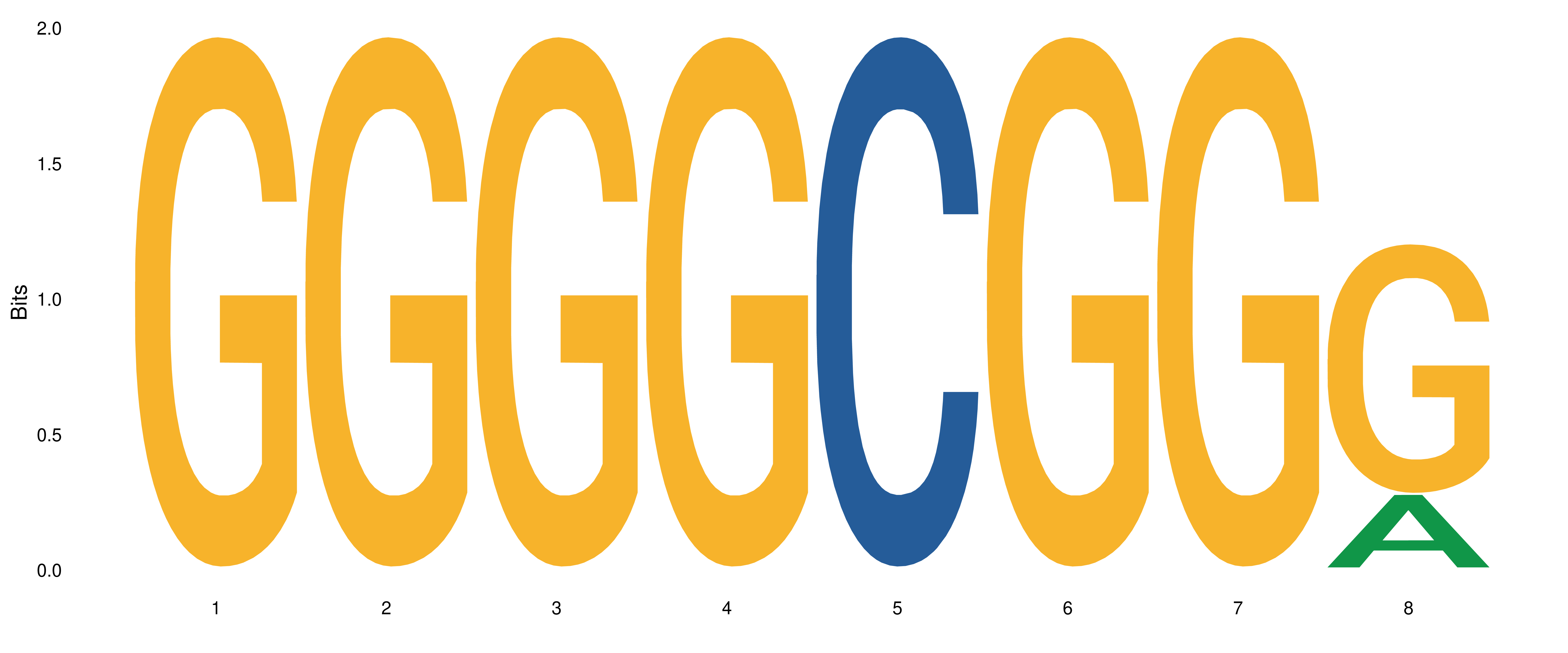 | 1e-22 | 40.66% | 21.84% | 426.1bp (808.6bp) |
| 2 | 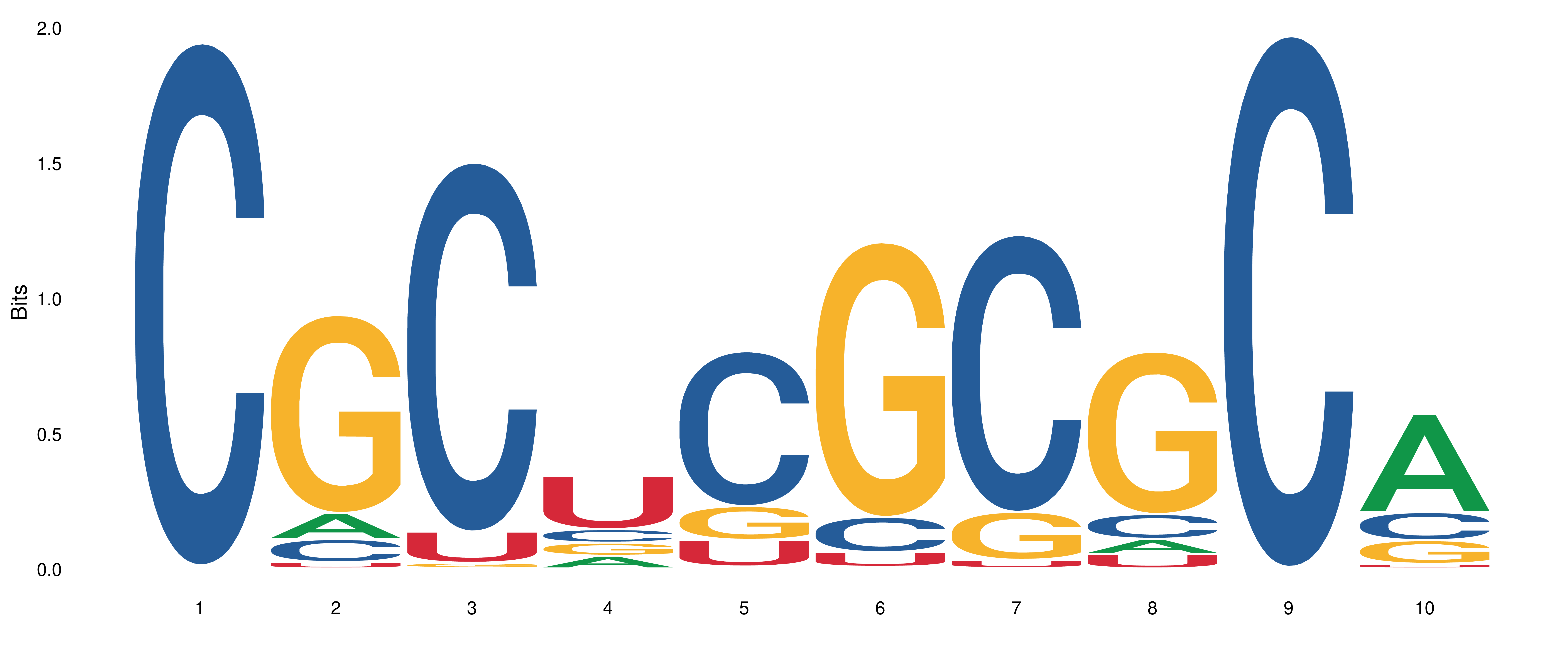 | 1e-16 | 37.91% | 21.98% | 448.4bp (848.0bp) |
| 3 | 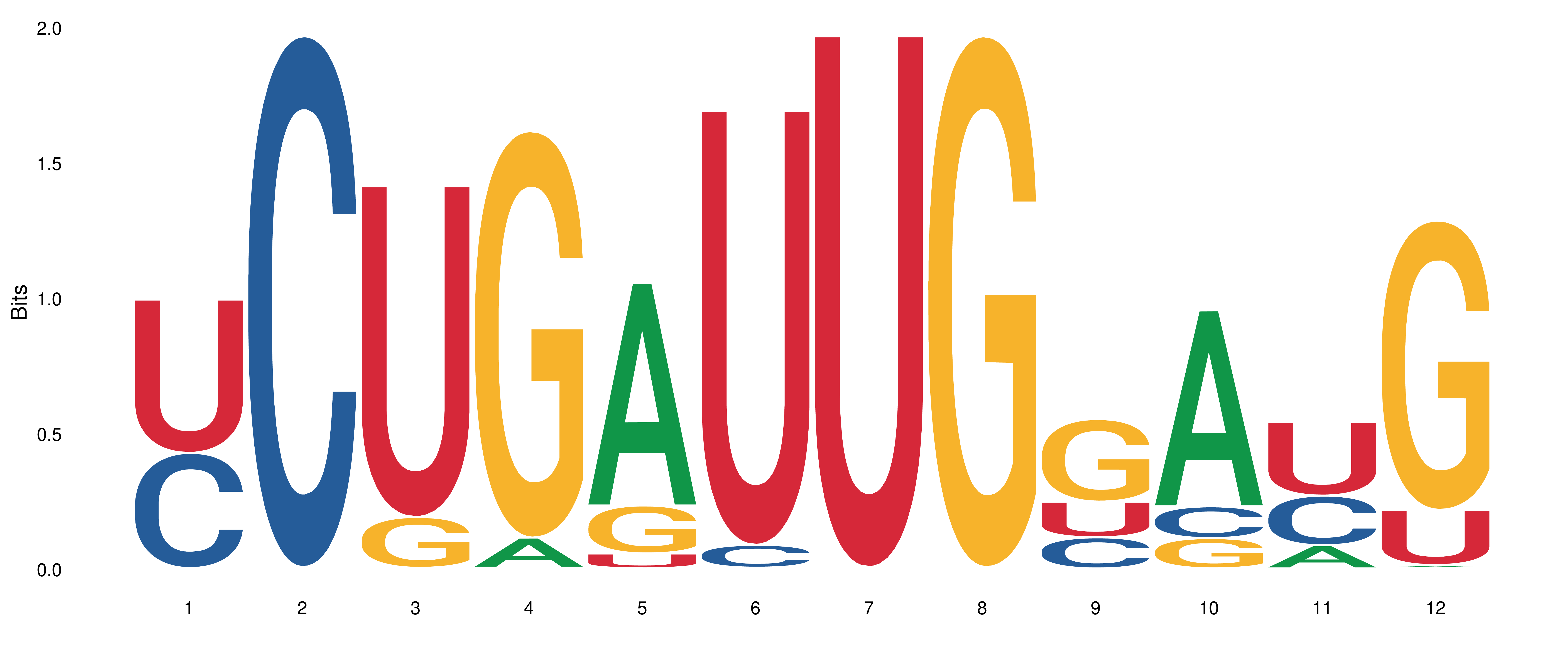 | 1e-13 | 5.86% | 1.06% | 595.4bp (638.0bp) |
| 4 | 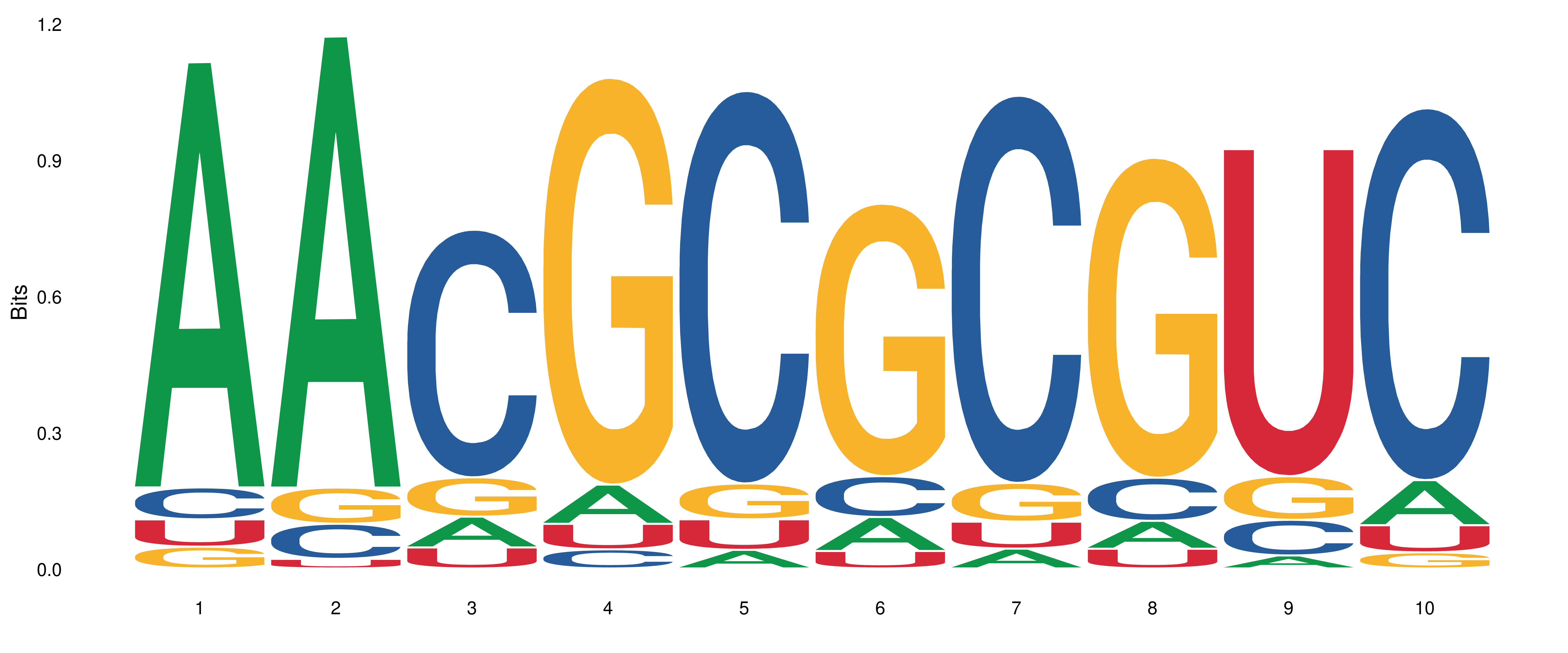 | 1e-13 | 33.88% | 20.01% | 527.8bp (784.4bp) |
| 5 | 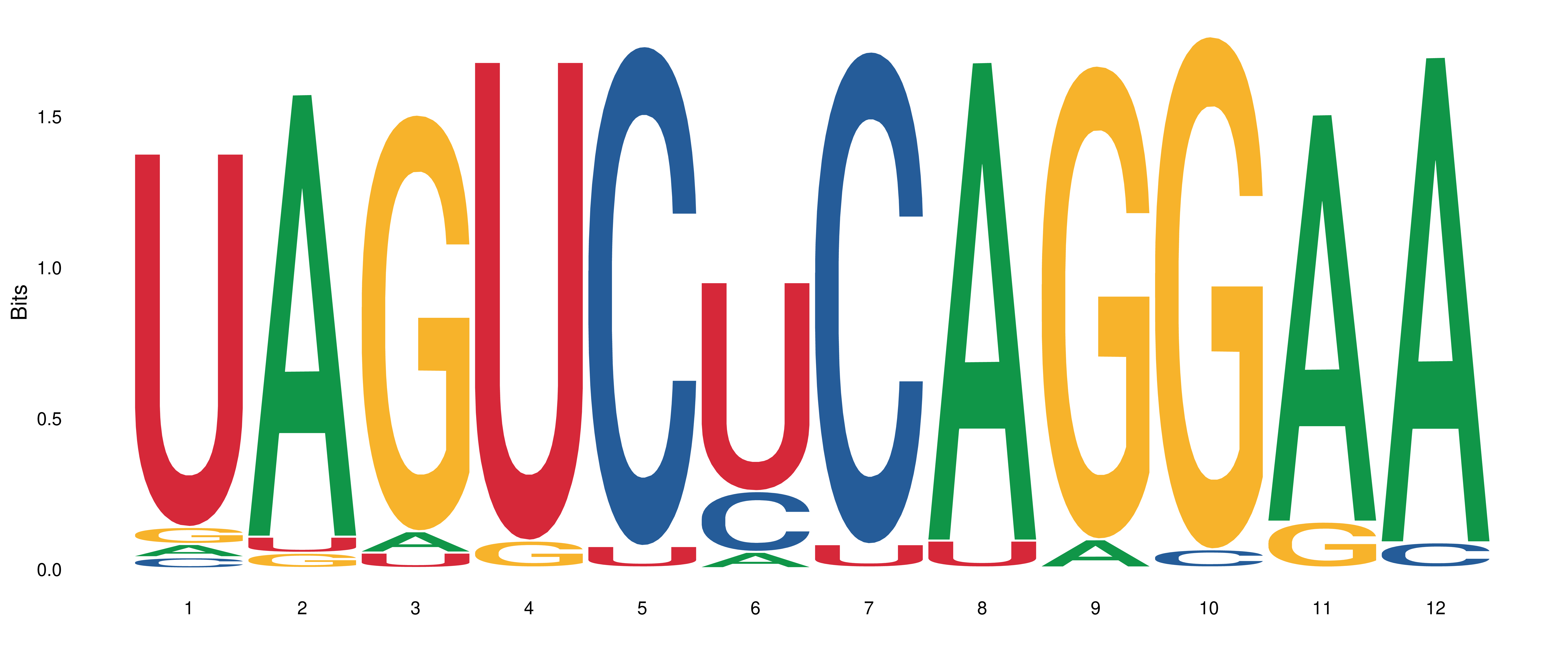 | 1e-11 | 4.03% | 0.58% | 458.5bp (582.5bp) |
| 6 | 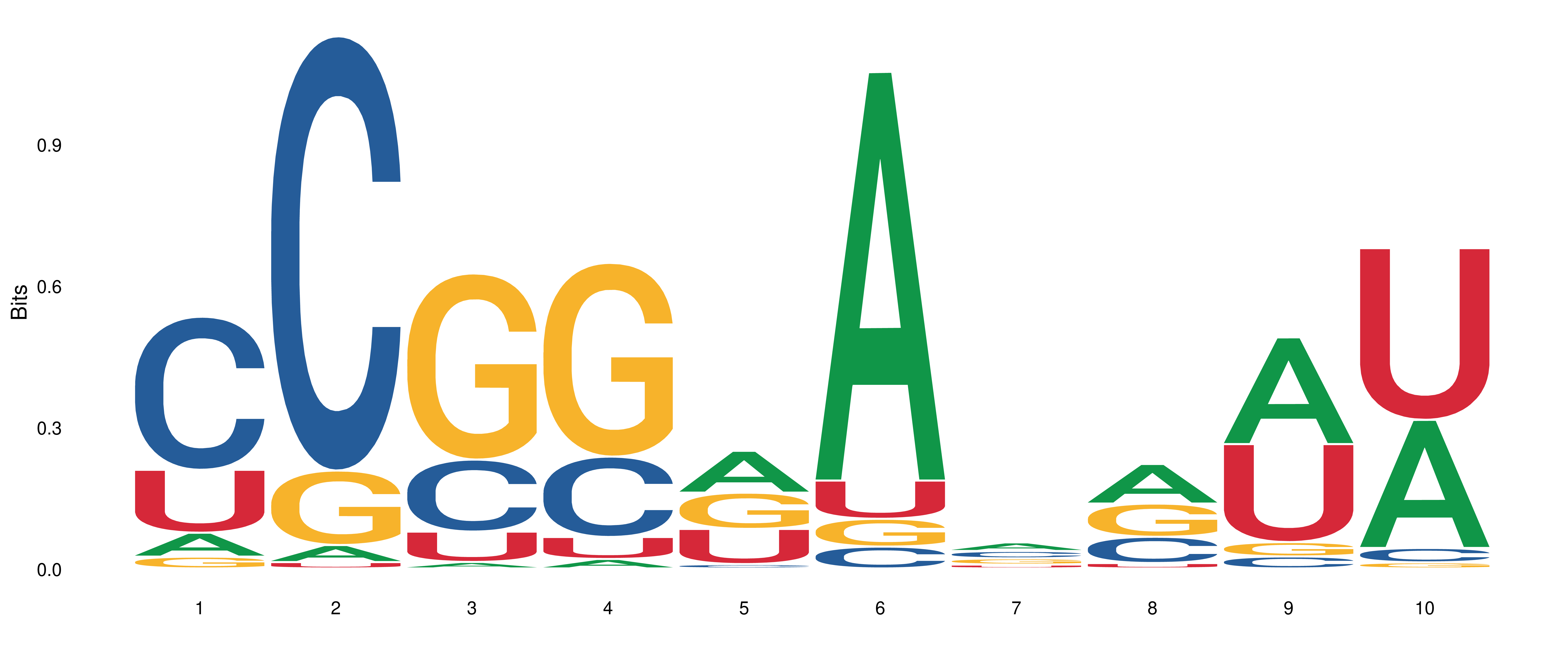 | 1e-11 | 12.09% | 4.68% | 591.7bp (706.7bp) |
| 7 | 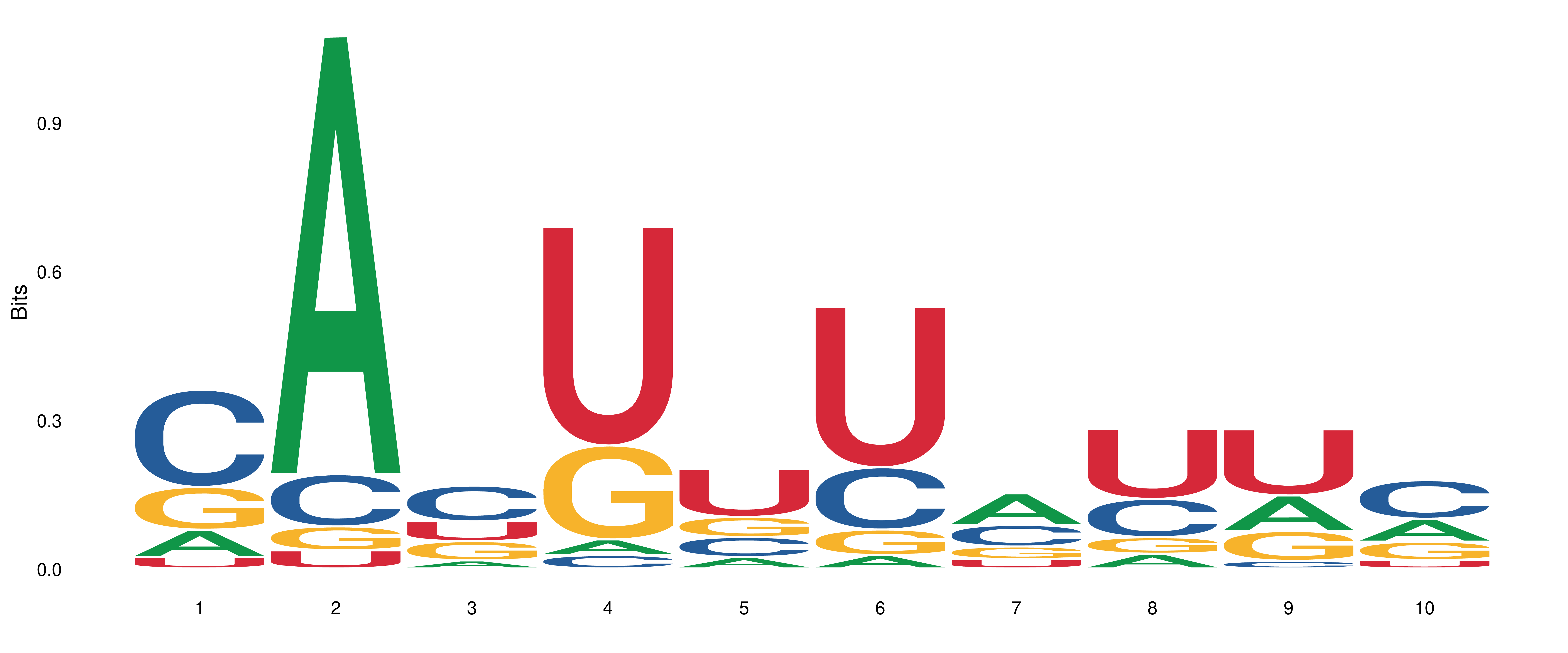 | 1e-11 | 16.85% | 7.98% | 521.3bp (622.9bp) |
| 8 | 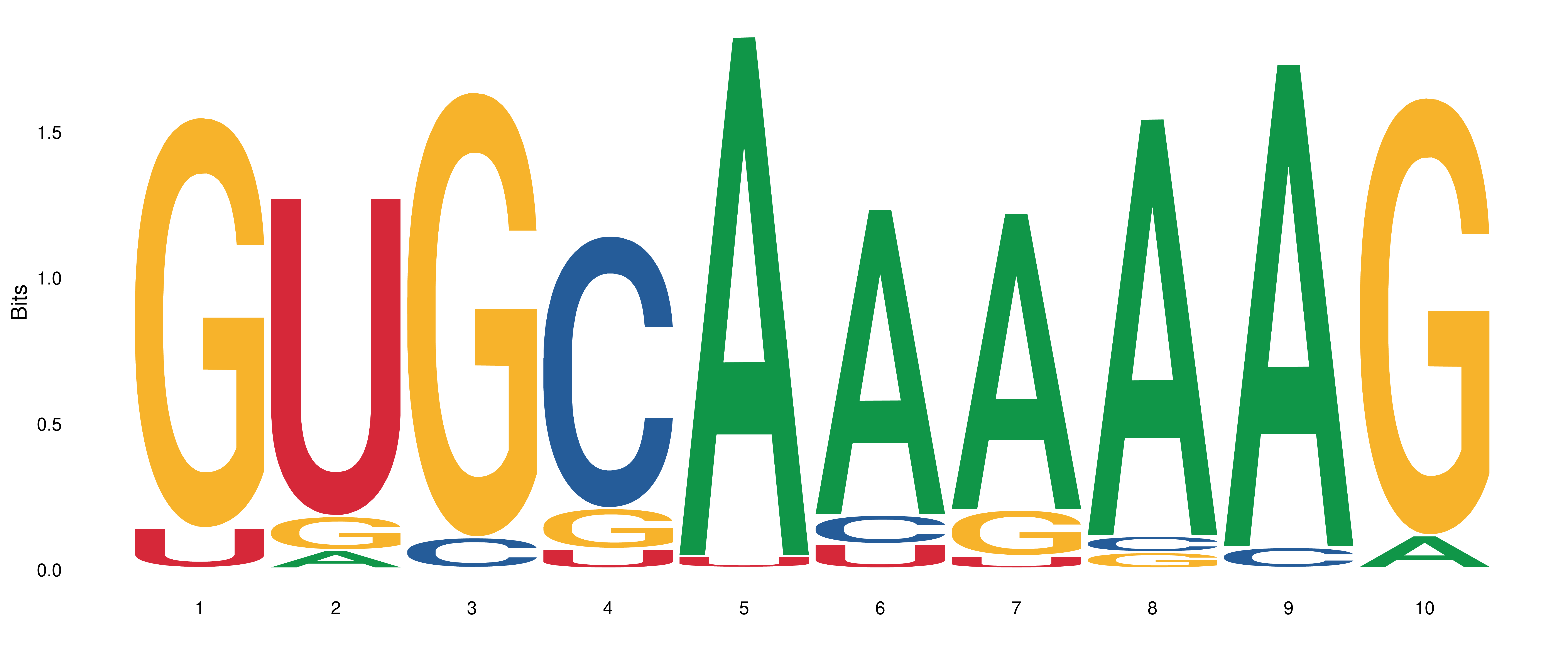 | 1e-9 | 14.29% | 6.54% | 573.3bp (636.2bp) |
| 9 | 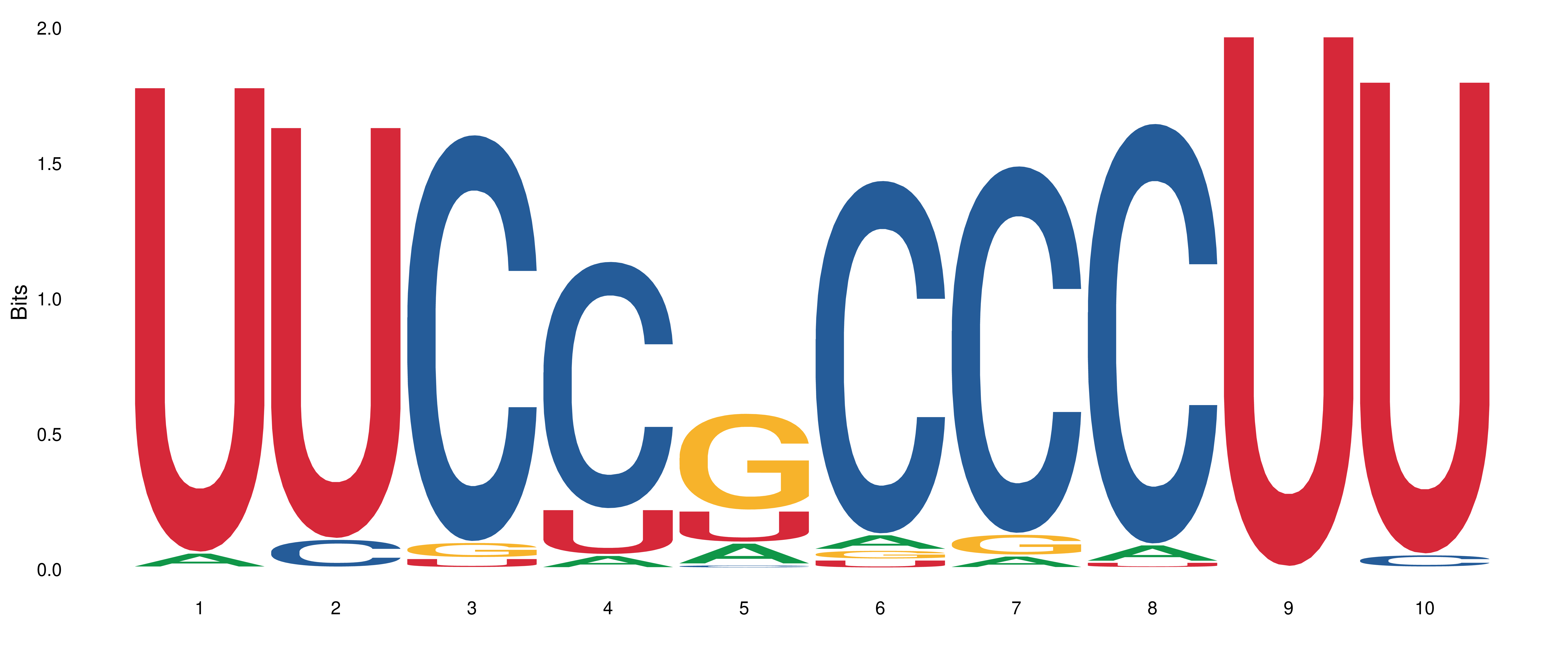 | 1e-9 | 17.22% | 8.67% | 611.4bp (637.3bp) |
| 10 | 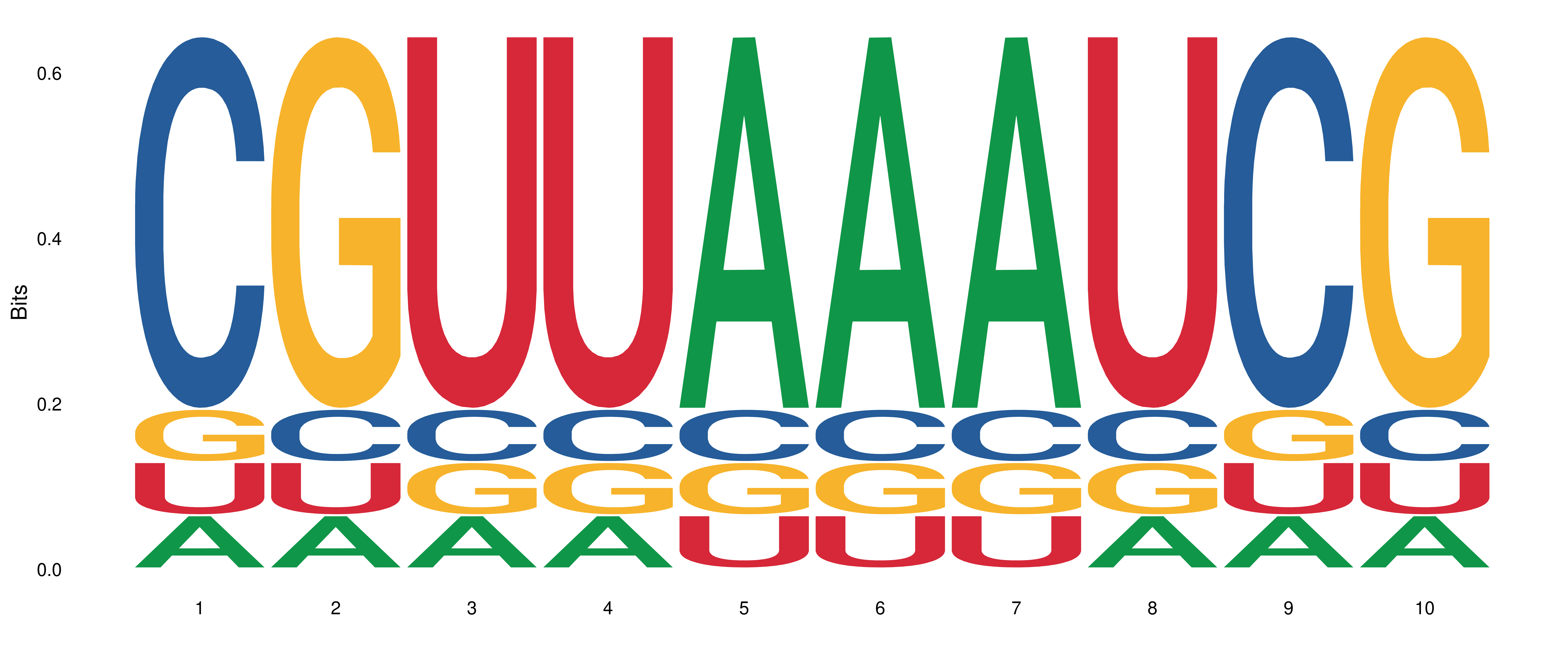 | 1e-9 | 0.73% | 0.00% | 423.0bp (203.1bp) |

% of Targets: The percentage of target sequences among all de novo identified sequences. % of Background: The percentage of background sequences among all de novo identified sequences. STD (Bg STD): Standard deviation of motif occurrence relative to the sequence center (within ±200 bp) in both target and background sets.
